# Excess labile carbon promotes the expression of virulence factors in coral reef bacterioplankton

**DOI:** 10.1038/ismej.2017.142

**Published:** 2017-09-12

**Authors:** Anny Cárdenas, Matthew J Neave, Mohamed Fauzi Haroon, Claudia Pogoreutz, Nils Rädecker, Christian Wild, Astrid Gärdes, Christian R Voolstra

**Affiliations:** 1 Leibniz Center for Tropical Marine Ecology (ZMT), Bremen, Germany; 2 Max Plank Institute for Marine Microbiology, Bremen, Germany; 3 Red Sea Research Center, Biological and Environmental Sciences and Engineering Division (BESE), King Abdullah University of Science and Technology (KAUST), Thuwal, Saudi Arabia; 4 Department of Organismic and Evolutionary Biology, Harvard University, Cambridge, MA, USA; 5 Marine Ecology Group, Faculty of Biology and Chemistry, University of Bremen, Germany

## Abstract

Coastal pollution and algal cover are increasing on many coral reefs, resulting in higher dissolved organic carbon (DOC) concentrations. High DOC concentrations strongly affect microbial activity in reef waters and select for copiotrophic, often potentially virulent microbial populations. High DOC concentrations on coral reefs are also hypothesized to be a determinant for switching microbial lifestyles from commensal to pathogenic, thereby contributing to coral reef degradation, but evidence is missing. In this study, we conducted *ex situ* incubations to assess gene expression of planktonic microbial populations under elevated concentrations of naturally abundant monosaccharides (glucose, galactose, mannose, and xylose) in algal exudates and sewage inflows. We assembled 27 near-complete (>70%) microbial genomes through metagenomic sequencing and determined associated expression patterns through metatranscriptomic sequencing. Differential gene expression analysis revealed a shift in the central carbohydrate metabolism and the induction of metalloproteases, siderophores, and toxins in *Alteromonas*, *Erythrobacter*, *Oceanicola*, and *Alcanivorax* populations. Sugar-specific induction of virulence factors suggests a mechanistic link for the switch from a commensal to a pathogenic lifestyle, particularly relevant during increased algal cover and human-derived pollution on coral reefs. Although an explicit test remains to be performed, our data support the hypothesis that increased availability of specific sugars changes net microbial community activity in ways that increase the emergence and abundance of opportunistic pathogens, potentially contributing to coral reef degradation.

## Introduction

Over the past decades, coral reefs have been rapidly degrading due to a combination of local and global factors that include overfishing (Jackson *et al.*, 2001), pollution (McCook, 1999), and climate change (Hughes *et al.*, 2003; Hoegh-Guldberg *et al.*, 2007), leading to an increase in coral bleaching and disease emergence (Weil *et al.*, 2006; Baker *et al.*, 2008). Although diseases represent an important cause of deterioration in these productive tropical marine communities, they are still poorly understood (Willis *et al.*, 2004; Weil *et al.*, 2006; Muller and Woesik, 2012). Paleontological and ecological monitoring suggest an increase in the prevalence of reef diseases over the last few decades (Harvell *et al.*, 1999; Porter *et al.*, 2001; Cramer *et al.*, 2012), partially as a consequence of nutrient enrichment (Bruno *et al.*, 2003; Voss and Richardson, 2006; Vega Thurber *et al.*, 2014), and particularly because of increases in dissolved organic carbon (DOC) (Kuntz *et al.*, 2005; Kline *et al.*, 2006; Smith *et al.*, 2006). Autochthonous DOC can enter coral reefs by coral and algal exudation (up to 40% of algal fixed carbon produced in photosynthesis) in the form of a heterogeneous mixture of saccharides, proteins, and lipids (Haas and Wild, 2010; Nelson *et al.*, 2013). Although exudates released by scleractinian corals contain high levels of refractory DOC and are therefore more similar in their sugar composition to reef waters and offshore DOC pools, turf and fleshy macroalgal-derived DOC is highly enriched in the labile sugars glucose, galactose, mannose, and xylose (Nelson *et al.*, 2013). Therefore, algae exudates are composed similarly to sewage discharge (Huang *et al.*, 2010; Wear and Vega Thurber, 2015). Owing to these qualitative differences, coral and macroalgal exudates differentially affect the composition and metabolism of coral reef seawater microbial communities (Nelson *et al.*, 2013; Haas *et al.*, 2016; Lee *et al.*, 2016). The detrimental effects of labile DOC on coral reef functioning are summarized in the DDAM (Disease, Dissolved organic carbon, Algae and Microbe) model (Barott and Rohwer, 2012).

The DDAM model proposes a positive feedback loop in which human-derived stressors, such as overfishing and eutrophication, trigger macroalgal growth and promote the release of DOC-enriched exudates (Haas *et al.*, 2013). This mostly labile DOC fosters bacterial growth and oxygen removal, selecting for copiotrophs and opportunistic pathogens both in the seawater and the coral holobiont (Vega Thurber *et al.*, 2009; Haas *et al.*, 2011; Nelson *et al.*, 2013; Haas *et al.*, 2016). This selection for potential pathogens in turn completes the loop as it promotes coral bleaching, disease, and eventually mortality, providing space for algae to grow and become dominant on the reef. The etiology of many coral diseases, however, is poorly understood. Although numerous coral diseases have been described, causative agents have been identified only in a few cases and with incomplete satisfaction of Koch's postulates (Richardson *et al.*, 1998; Alker *et al.*, 2001; Sutherland *et al.*, 2011). Efforts towards the characterization of the whole microbial community rather than looking for single agents have made significant advances in the field. For instance, the appearance of signs of coral disease often correlates with shifts in coral-associated bacterial community composition (Sunagawa *et al.*, 2009; Cárdenas *et al.*, 2012; Roder *et al.*, 2014), suggesting a more complex pathogenesis. To date, these diseases are believed to be opportunistic infections triggered by the exposure to environmental stressors (for example, elevated temperature or nutrient enrichment), reducing host resistance and promoting the uncontrolled growth of opportunistic pathogens (Landsberg, 1995; Rosenberg *et al.*, 2007). These opportunistic pathogens can originate either from the coral holobiont itself (symbionts) or the water column (environmentally acquired) and are also associated with healthy reefs, where they fulfill key functions to support the ecosystem, for example, antibiotic production and nitrogen fixation (Ritchie, 2006; Chimetto *et al.*, 2008; Mohamed *et al.*, 2008).

Virulence can be understood as harm or morbidity caused by a microbe to its host. Thus, identifying virulence determinants or factors (VF) can provide insights into the mechanistic basis of such harm (Brown *et al.*, 2012). VFs are molecules produced by pathogens that contribute to pathogenicity and typically include toxins, exoenzymes, adhesins, and secretion systems (Brown *et al.*, 2012). The expression of VFs is commonly correlated with the ability of a pathogen to invade and exploit host tissues. Nelson *et al.* (2013) showed that algal exudates support the growth of bacterioplankton populations among the families Vibrionaceae and Pseudoalteromonadaceae harboring high numbers of VFs. Temperature-dependent expression of virulence factors has been reported in several *Vibrio* species (Banin *et al.*, 2003; Kimes *et al.*, 2012). Despite these studies, there has been no compelling evidence correlating other environmental factors with the activation of VFs in opportunistic coral reef pathogens.

Expression of VFs could provide a selective advantage for opportunistic microbes to gain access to nutrients. Therefore, these genes are often linked to general pathways of nutrient uptake (Görke and Stülke, 2008). As an example, so-called moonlighting proteins are involved in major metabolic processes such as the glycolytic pathway and stress responses that have unexpected functions which contribute to microbial virulence (Henderson and Martin, 2011). Carbon catabolism is linked to bacterial virulence by the sensing of sugars through phosphotransferase system transporters, which in turn activate carbon catabolite repression (Deutscher *et al.*, 2006; Görke and Stülke, 2008). When different carbon sources are available, carbon catabolite repression allows bacteria to control the uptake of the preferred carbon substrate by disabling genes involved in the use of secondary carbon sources (Brückner and Titgemeyer, 2002). Carbon catabolite repression is observed in most free-living heterotrophic bacteria and acts as a global regulator, controlling up to 10% of all bacterial genes, including several virulence factors (Yoshida *et al.*, 2001; Blencke *et al.*, 2003; Görke and Stülke, 2008). The combination of carbon catabolite repression and multiple transcriptional regulation networks allows copiotrophic bacteria to succeed at high-carbon concentrations (Cottrell and Kirchman, 2016).

A prediction of our pathogen emergence model is that elevation of specific sugars will change the microbial community composition and microbial gene expression, thereby promoting the risk of reef degradation. Therefore, in the present work, we characterized the response of microbial communities from coral reef seawater samples before and after the addition of the dominant monosaccharides previously reported for algal exudates and sewage discharge (glucose, galactose, mannose, xylose). This was achieved in an *ex situ* approach by amending natural coral reef seawater samples in single monosaccharide incubations in the dark in 4 L microcosms over 48 h. Microbial communities were characterized via 16 S rRNA gene amplicon sequencing and reef planktonic population genomes were recovered via metagenomic binning. Microbial mRNA reads were subsequently mapped to the recovered population genomes to assess the activity of distinct microbial community members under sugar enrichment. This allowed us to elucidate potential mechanisms that suggest negative interactions between planktonic microbes and corals driven by high sugar concentrations.

## Materials and methods

### Water sampling and incubations

Incubations consisted of three liters of 0.22 μm-filtered seawater inoculated with one liter of unfiltered reef seawater in a four-liter polycarbonate bottle (sulfuric acid-cleaned and seawater-leached). Unfiltered seawater was collected at 10 m depth from Al-Fahal reef located 13 km offshore in the central Red Sea of Saudi Arabia at 9:30 am on 20 October 2014 and 03 November 2014 for the first and second set of experiments, respectively. Every experiment consisted of four triplicate treatments, two sugar-amended and two unamended controls. Experiment 1 consisted of (B1) unamended control before incubation, (C1) unamended control after incubation, (Glu) after glucose incubation and (Gal) after galactose incubation. Experiment 2 consisted of (B2) unamended control before incubation, (C2) unamended control after incubation, (Man) after mannose incubation and (Xyl) after xylose incubation. Each treatment was amended with 500 μm of the corresponding sugars. The concentration was chosen to elicit rapid microbial responses. The incubation time was 48 h at 27 °C in the dark to avoid phytoplankton growth. Supplementary Figure 1 shows a schematic representation of the different experiments and enrichments.

### Cell density, DOC concentrations, and efficiency calculations

Changes in cell abundance and DOC concentrations were measured over time during sugar incubations. Bacterial cell abundance was determined by flow cytometry using the protocol established by Nelson *et al.* (2011). In brief, unfiltered samples were fixed with paraformaldehyde to a final concentration of 0.4% and frozen (−80 °C) within 30 min of fixation. Fixed samples were thawed, mixed, and stained with 1 × SYBR Green for 1 h at room temperature in the dark. Cell counts were done using the Guava easyCyte flow cytometer (Millipore, Billerica, MA, USA). To measure DOC concentrations, triplicates of 20 ml samples were filtered through 0.45 μm pore size Minisart-GF filters (Sartorius, Gottingen, Germany). The filtrate was collected in 25 ml pre-rinsed polyethylene HDPE bottles. A total of 100 μl of a 35% H_3_PO_4_ solution were added to acidify the samples (pH below 2) and subsequently stored at −20 °C in the dark until processing. DOC concentrations were measured in triplicates using the Apollo 9000 total organic carbon and Total Nitrogen Analyzer (Teledyne Tekmar, United States) at the Analytical Core Lab at KAUST, Saudi Arabia. Bacterial carbon change was determined as the difference in cell carbon (μmol C l^−1^) before and after the incubation using a previously reported factor for coastal bacteria assemblages of 30 fg C per cell (Fukuda *et al.*, 1998). Bacteria growth efficiency was calculated as the ratio of bacterial carbon production (rate of increase in bacterial carbon) to the rate of DOC removal (Eiler *et al.*, 2003).

### Nucleic acid extraction and isolation

Triplicate water samples were pre-filtered subsequently through 10 and 3 μm polycarbonate filters and collected onto 0.22 μm polycarbonate filters. Filters were immediately frozen at −80 °C until nucleic acid extraction. Replicate filters were pooled for nucleic acid extractions using the AllPrep DNA/RNA kit (Qiagen, Hilden, Germany). Purified DNA was used for rRNA gene amplification and preparation of metagenomic sequencing libraries (see below).

About 200 ng of total RNA were amplified using the MessageAmp II-Bacteria kit (Ambion, Austin, TX, USA) as described previously (Frias-Lopez *et al.*, 2008; Shi *et al.*, 2011). Prokaryotic mRNA was purified according to Stewart *et al.* (2010) with slight modifications (Daniels *et al.*, 2015). In brief, rRNA was subtracted from the total RNA using anti-sense probes created by the *in vitro* transcription of ribosomal genes (bacterial and archaeal 16S/23S and eukaryotic18S/28S) at a final template-to-probe ratio of 1:2 (mass, per probe). Subsequently, mRNA was subjected to an oligodT-based separation to exclude polyA+ eukaryotic mRNA from the polyA- prokaryotic mRNA. Metatranscriptomic TrueSeq libraries were sequenced using the Illumina MiSeq V3 system (Illumina, San Diego, CA, USA) with paired-end reads (2 × 300 bp). Metagenomic TrueSeq libraries were sequenced using the Illumina HiSeq 2000 system (Illumina) with paired-end reads (2 × 150 bp) according to the manufacturer's specifications.

### 16S-based diversity analysis

Microbial community composition was assessed via 16S rRNA sequencing before and after sugar enrichments. For this, 16S rRNA V5-V6 hypervariable regions were PCR-amplified for 25 cycles using 16S primers 784 F (5′-AGGATTAGATACCCTGGTA-3′) and 1061 R (5′-CRRCACGAGCTGACGAC-3′) designed by Andersson *et al.* (2008), which work well in marine environments (Bayer *et al.*, 2013). PCR products were sequenced using Illumina MiSeq V3 (2 × 300 bp) according to the manufacturer’s specifications at the Bioscience Core Lab (KAUST, Saudi Arabia). Primer trimming, quality control, clustering and taxonomic classification were done in mothur v1.36.1 (Schloss *et al.*, 2009). Quality control consisted of removing sequences shorter than 300 bp, and/or with quality scores lower than 25 and those that occurred only once (singletons) across the entire dataset. Sequences were aligned to SILVA reference, release 119 (Quast *et al.*, 2013), and clustered into operational taxonomic units (OTUs), defined at 97% similarity. Representative sequences from each cluster were taxonomically assigned using Greengenes reference taxonomy (McDonald *et al.*, 2012). Chimeric sequences were identified with the *de novo* implementation of UCHIME in the mothur interface (Edgar *et al.*, 2011) and subsequently removed. Principal Coordinate Analysis using Bray-Curtis distance and diversity analyses (Shannon-Weaver and Chao1 indexes) was performed on operational taxonomic unit abundance data using the Phyloseq (McMurdie and Holmes, 2013) R package, using output tables generated in mothur.

### Metagenome binning

Sequence reads were quality checked and trimmed for low-quality regions, adapter sequences, and a minimum length of 75 bp using Trimmomatic v0.36 (Bolger *et al.*, 2014). MEGAHIT v1.0.2 (Li *et al.*, 2015) was used to produce one assembly per experiment using the ‘meta-sensitive’ option and *kmer* length of 81. Metagenome binning was done based on differential coverage and tetranucleotide signatures for each assembly separately using the binning tools GroopM v0.3.4 (Imelfort *et al.*, 2014) and Metabat v0.25.4 (Kang *et al.*, 2015). Genome contamination and completeness were assessed using CheckM v1.0.3 (Parks *et al.*, 2015). To increase genome completeness, the ‘merge’ option from CheckM was used and population genomes with a delta contamination lower than 3 and a merged contamination lower than 5 were merged. CheckM ‘unique’ and ‘join’ commands were used to compare and combine genomes generated from the different binning tools. The metagenomic binning procedure resulted in population genomes representing putative microbial taxa. A first taxonomic approximation was done based on universal, single-copy phylogenetic marker genes using specI (Mende *et al.*, 2013), marker lineages from CheckM, and sequence composition-based classifier (Patil *et al.*, 2012). Whole-genome based average nucleotide identity and amino-acid identities between the query genomes and NCBI reference genomes were used to validate closest related taxa using JSpecies v1.2.1. (Richter and Rosselló-Móra, 2009) and the enveomics tools (Rodriguez-R and Konstantinidis, 2016). Open reading frames were predicted using Prodigal (Hyatt *et al.*, 2010). Predicted protein-encoding genes were functionally annotated using Gene Ontology (GO) and myRAST server (Aziz *et al.*, 2008) according to SEED subsystems (Overbeek *et al.*, 2014). Specific processes were inferred from GO annotation as listed in the supplement (Supplementary Table S2). For instance, microbial interaction genes were identified based on the PAMGO (Plant-Associated Microbe Gene Ontology) Consortium (Torto-Alalibo *et al.*, 2009). Virulence genes were assigned by a BLASTn v2.2.28 search against MvirDB (Zhou *et al.*, 2007) and VFDB (Chen *et al.*, 2005) databases (both downloaded on 25 March 16), supplemented with GO terms related to pathogenesis (Supplementary Table S2). Sugar transporters were identified using the Carbohydrate Active Enzymes database (http://www.cazy.org/) (Cantarel *et al.*, 2009). Genes involved in antibiotic resistance were deducted from the BLAST search against the Antibiotic Resistance Genes Database (ARDB) (downloaded on 07 July 16) (Liu and Pop, 2009).

### RNA mapping and gene expression analysis

Remaining rRNA in metatranscriptomic data were removed *in silico* using SortMeRNA v2.0 (Kopylova *et al.*, 2012). Cleaned mRNA fragments were mapped to a multifasta file containing all annotated genomes using the Bowtie aligner v1.0.0 using the ‘best’ option allowing a single hit (Langmead and Salzberg, 2012). Transcript-level estimates were obtained as mRNA read counts using the transcript abundance estimation tool eXpress v1.5.1 (Roberts and Pachter, 2013) and normalized by the size of the RNA library (sequencing depth) and the coverage of the population genome in the metagenome (population genome abundance). Differential expression, expressed as log2 fold change, was calculated for mRNA reads in each treatment compared with the controls (that is, C1 and C2 samples). A twofold fold change cutoff was used to determine up- and downregulated genes. Upregulated genes (that is, induced genes) were used to establish changes in biological processes after sugar addition.

### Data deposition

Raw nucleotide sequences are available from the NCBI Sequence Read Archive under BioProject accession number PRJNA352340 (https://www.ncbi.nlm.nih.gov/bioproject/PRJNA352340/).

## Results and discussion

The response of reef seawater microbial communities to labile DOC enrichment was investigated to understand the complex interactions and mechanisms involved in the emergence of pathogenicity in these communities. The monosaccharide additions to *ex situ* microcosms elicited differential, sugar-specific responses reflected in rapid microbial community shifts, gene expression, and associated virulence potential.

### Microbial diversity and DOC consumption

**Figure 1:**
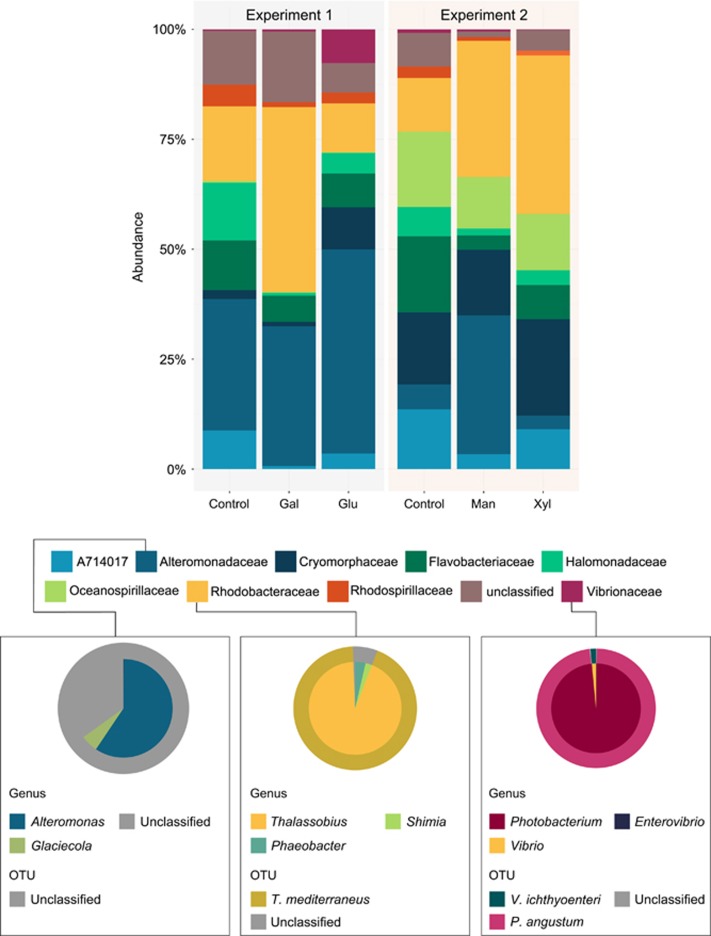
Impact of high sugar concentrations on community structure of reef planktonic microbial incubations. Bar charts show the relative abundance of OTUs in reef water samples before and after addition of different sugars. Pronounced shifts of Alteromonadaceae, Rhodobacteraceae, and Vibrionaceae are evident. Outer and inner rings on the pie charts show taxonomic affiliation of these OTUs at species and genus level, respectively. Gal, Galactose, Glu, Glucose, Man, Mannose, Xyl, Xylose.

The main differences in operational taxonomic unit (OTU) composition between both controls (natural reef water, no sugar added, not incubated) were a higher relative abundance of Alteromonadaceae and lower Oceanospirillaceae in experiments 1 compared with experiment 2. These differences were likely due to small changes in inorganic nutrient concentrations driven by seasonality (Roik *et al.*, 2016) as well as by differences in DOC concentrations in the natural reef water before the start of the experiments (Table 1). After 48 h incubations, sugar-enriched samples deviated from the controls in a sugar-specific manner, generally towards a less-rich and less-diverse bacterial community (Supplementary Figure S2A and B). Microbial community shifts were characterized by increases in Rhodobacteraceae (Alphaproteobacteria) in galactose, mannose, and xylose, Vibrionaceae (Gammaproteobacteria) in glucose, and Alteromonadaceae (Gammaproteobacteria) in both glucose and mannose enriched samples (Figure 1). A recent study supporting the microbialization concept showed that oligotrophic coral-dominated waters are characterized by high abundance of Alphaproteobacteria, in contrast to enriched algal-dominated waters where Gammaproteobacteria are abundant (Haas *et al.*, 2016). Linking our study to Haas *et al.* (2016), our results suggest glucose and mannose are major contributors to bacterial shifts in impacted reefs. Furthermore, microbial communities had significantly faster and more efficient growth in galactose and glucose enrichments (Table 1) suggesting that these sugars play a major role in supporting an increase in microbial biomass. However, efficiency estimates can be influenced by the conversion factor for coastal bacteria assemblages of 30 fg C per cell used here. This is only a rough estimate, and human impact and increased algal cover can also significantly influence cell size (McDole *et al.*, 2012).

### Metagenomic binning and microbial population genomes

We assembled metagenomic reads from each experiment and calculated the abundance profile and tetranucleotide frequencies of each contig between treatments. This allowed us to assign co-abundant contigs with similar tetranucleotide frequencies into groups that represent the gene content of distinct microbial population genomes representing putative microbial taxa. We recovered >400 draft population genomes and only used those with total completeness above 70% for further analyses (Table 2). Annotation and gene expression analyses were conducted for the selected 27 population genomes, after determining nucleic acid and amino-acid identities of complete reference genomes to infer closest related organisms (Supplementary Table S3). Population genomes comprised a wide diversity of taxa including different populations of the copiotrophic *Alteromonas* and several members of the Rhodobacteraceae, such as *Oceanicola*, *Ruegeria*, and *Labrenzia* (Table 2).

**Figure 2:**
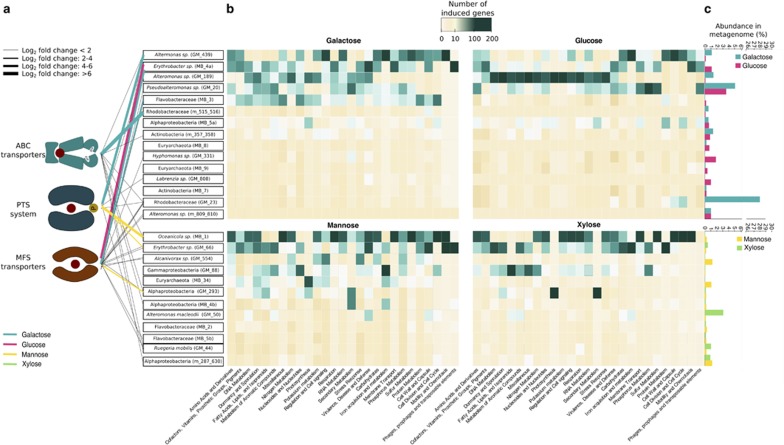
Sugar transporter diversity and expression in microbial population genomes. (**a**) Expression of sugar transporters across microbial population genomes. The expression of sugar transporters (ABC, PTS, or MFS) is represented in lines linked to each population genome. Fold changes <2 (that is, not induced) are denoted in black thin lines. Thicker lines represent fold changes>2 (that is, induced) after enriching with sugars colored in blue for galactose, magenta for glucose, yellow for mannose, and green for xylose. (**b**) Heat map depicting the number of induced genes across the population genomes in biological processes using SEED subsystems annotation. Bacterial population genomes response is clustered by Euclidian distance for galactose and mannose. (**c**) Relative abundance of each microbial population genome in the metagenome, calculated as the proportion of metagenomic reads mapped to the population genome from the total number of reads in the metagenomic library.

To profile transcriptomic patterns in planktonic microbial populations, bacterial mRNA sequences were mapped to the recovered population genomes. Mapping counts were normalized to the genome abundance in the overall population, thereby uncovering gene expression at an individual rather than population level. Furthermore, the results of the present work are focused on differentially upregulated genes (that is, induced genes) after comparing expression levels in the control versus each treatment. Populations with high numbers of differentially expressed genes were identified using RAST annotation (Aziz *et al.*, 2008) of induced biological processes (Figure 2b and Supplementary Figure S3). These population genomes included members of the Gammaproteobacteria genera *Alteromonas, Pseudoalteromonas, Alcanivorax*, and *Hahella*, and of the Alphaproteobacteria genera *Oceanicola* and *Erythrobacter*. Most of these are comprised of microbes typically found in coral reefs (Frias-Lopez *et al.*, 2002; Bourne and Munn, 2005; Tout *et al.*, 2015), but are often linked to nutrient-enriched and degraded reefs including fish farm effluents (Garren *et al.*, 2008), oil-contaminated reefs (Al-Dahash and Mahmoud, 2013), and coral diseases (Sunagawa *et al.*, 2009; Cárdenas *et al.*, 2012; Roder *et al.*, 2014).

### Sugar transporter diversity and microbial metabolism

The recovery of near-complete population genomes allowed us to investigate cellular mechanisms activated by the sensing and uptake of dissolved monosaccharides. The first step in understanding sugar uptake was to evaluate the diversity of sugar transporters. The most common transporters among the population genomes were galactose ABC transporters, mannose phosphotransferase system, and glucose/mannose major facilitator superfamily porters. The mannose phosphotransferase system is not only an important uptake system for mannose and glucose, but it has a major role in the transcriptional regulation and the preferential utilization of monosaccharides (Postma *et al.*, 1993; Vu-Khac and Miller, 2009). The phosphotransferase system transporters were highly induced in population genomes with higher numbers of differentially expressed genes, specifically genes involved in symbiotic interactions and virulence factors (discussed below) (Figures 2a and b). Copiotrophs were expected to possess a wide array of low-affinity and highly specific transporters, contrary to oligotrophs that have a smaller number of broad-specificity and sufficiently high-affinity transporters (Lauro *et al.*, 2009). This was confirmed in the most abundant population genomes (Figure 2c), such as GM_23 (Rhodobacteraceae), which possessed five different transporters for sugar uptake (Figure 2a). Most of these transporters belonged to the low-affinity ABC transporters and the multi-sugar major facilitator superfamily, whereas high-affinity transporters were rare. The presence of several glucose uptake systems may allow for flexibility under different environmental conditions. For instance, low-affinity systems are expected to be induced in sugar-rich environments. Contrastingly, high-affinity transporters are used when low amounts of glucose are available (Jahreis *et al.*, 2008).

**Figure 3:**
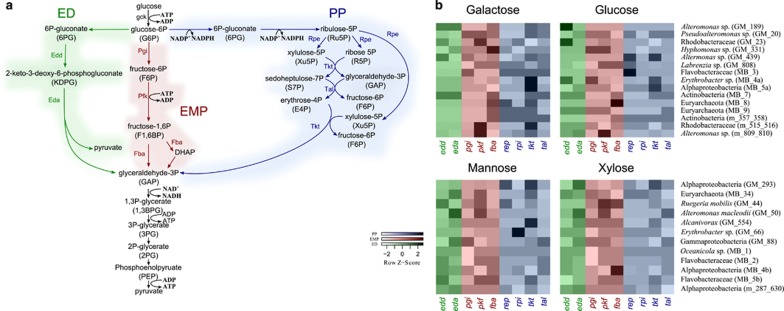
Expression of genes involved in different glycolytic pathways. (**a**) Entner–Doudoroff (ED) in green, Embden–Meyerhof–Parnas (EMP) in red, and pentose phosphate pathways (PP) in blue. (**b**) Each row represents a population genome and each column represents a distinct gene. Low expression is denoted by low intensity colors and high expression by high intensity colors. Expression levels correspond to log2 fold changes comparing control and treatment of sequencing depth- and bin coverage-normalized mRNA counts.

Using metagenomics, Haas *et al.* (2016) found that coral-dominated reef waters were enriched for genes in the energy efficient EMP (Embden–Meyerhof–Parnas) pathway, whereas algal-dominated reefs waters were enriched with genes in the less-efficient ED (Entner–Doudoroff) and PP (pentose phosphate) pathways. To test whether we saw a similar pattern, we evaluated the expression of genes involved in the EMP, ED, and PP pathways in the population genomes (Figure 3). We chose to focus on the pathway with highest expression levels in each of the population genomes. Although the response was sugar- and population-specific, some general patterns could be established. For instance, population genomes MB_8 (Euryarchaeota), merged_809_810 (*Alteromonas*), and MB_4b (Alphaproteobacteria) had highest expression levels of the EMP genes. However, genes involved in the ED and PP pathways were induced in population genomes GM_189 (*Alteromonas*), MB_4a (*Erythrobacter*), GM_554 (*Alcanivorax*), GM_293 (Alphaproteobacteria) and MB_66 (*Erythrobacter*). For the latter population genomes, we could corroborate previous findings that support shifts from EMP to ED and PP. These shifts in the metabolic capacities were suggested to represent a less costly, faster, but less-efficient transformation of organic carbon under high-carbon availability (Flamholz *et al.*, 2013; Haas *et al.*, 2016).

### Induction of microbial interaction genes

In general, microbial symbioses can be beneficial, neutral, or pathogenic (Hillman and Goodrich-Blair, 2016). Microbial interaction genes comprise several mechanisms by which microbes colonize, grow in, and occasionally cause detrimental effects to hosts and host tissues (Dale and Moran, 2006). Interaction genes were classified into distinct categories including biofilm formation, adhesion, secretion systems, and genes involved in pathogenesis (among others), according to the PAMGO consortium (Torto-Alalibo *et al.*, 2009). Numerous interaction genes identified in the population genomes belonged to antibiotic transport and production (Supplementary Figure S4). Antibiotic-producing bacteria often associate with marine organisms (Kennedy *et al.*, 2009; Nissimov *et al.*, 2009; Wiese *et al.*, 2009), and consequently, coral mucus-associated bacteria are considered the first line of defense (Ritchie, 2006; Shnit-Orland and Kushmaro, 2009). Contrastingly, free-living bacteria are irregular antibiotic producers (Nair and Simidu, 1987; Long and Azam, 2001). Therefore, exhibiting this trait could represent an advantageous strategy to colonize and effectively outcompete native benthic bacteria in new habitats. Another important group of interaction genes, ‘viral processes’, included viral–host interactions (Supplementary Figure S4). The latter suggests that viral infections are also playing major roles driving bacterioplankton community diversity in this study, as in other marine ecosystems (Middelboe *et al.*, 2001; Weinbauer and Rassoulzadegan, 2004; Holmfeldt *et al.*, 2007; Vega Thurber *et al.*, 2017).

**Figure 4:**
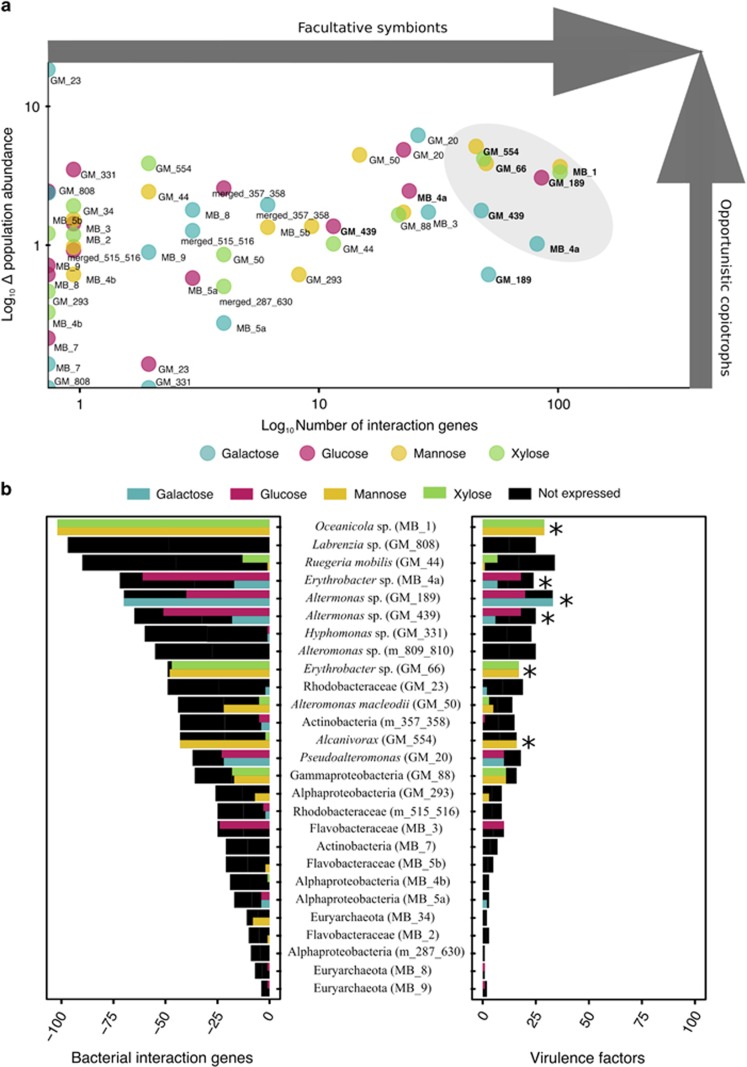
Interaction and virulence factor genes in microbial population genomes. (**a**) Change in population abundance between control and treatments (metagenome, *y* axis) in relation to the differentially expressed bacterial interaction genes (metatranscriptome, *x* axis). Increasing numbers on the *y* axis represent organisms with higher changes in abundance after sugar addition, whereas increasing numbers on the *x* axis denote bacterial species with a high number of expressed interaction genes. Population genomes that are more likely to initiate host interactions (that is, bacteria that grow rapidly under sugar enrichment, expressing a high number of interaction genes) are highlighted in a gray area. (**b**) Black bars represent the total number of interaction genes (left) and virulence factors (right) present in each population genome. Colored bars represent the number of differentially expressed genes under either galactose (blue), glucose (magenta), mannose (yellow), or xylose (green) enrichments. Stars denote population genomes with the largest number of virulence factors and correspond to the selected population genomes from (A).

A large number of interaction genes were categorized as virulence factors owing to their high homology with previously reported virulence factors in other microbial species. Virulence factors represent the subset of interaction genes that include all genes involved in negative microbial interactions according to PAMGO (Torto-Alalibo *et al.*, 2009) and MvirDB (Zhou *et al.*, 2007). We classified the population genomes based on their abundance in the metagenome and the highest number of induced interaction genes and virulence factors into the following microbial lifestyles: (1) the larger the increase in abundance after sugar enrichment, the more likely to have a copiotrophic lifestyle (Figure 4a), (2) the larger the number of interaction genes, the more likely this population genome represents an opportunistic symbiont (Figures 4a and b), (3) the larger the number of virulence factors, the more likely this population genome represents an opportunistic pathogen (Figure 4b). Based on these criteria, we chose the following six opportunistic pathogens with copiotrophic lifestyles and defined them as Potential Opportunistic Pathogens (POPs): MB_1 (*Oceanicola* sp.), GM_189 (*Alteromonas*), GM_439 (*Alteromonas*), GM_554 (*Alcanivorax*), MB_4a (*Erythrobacter*), and GM_66 (*Erythrobacter*).

### Virulence potential in opportunistic pathogens

**Figure 5:**
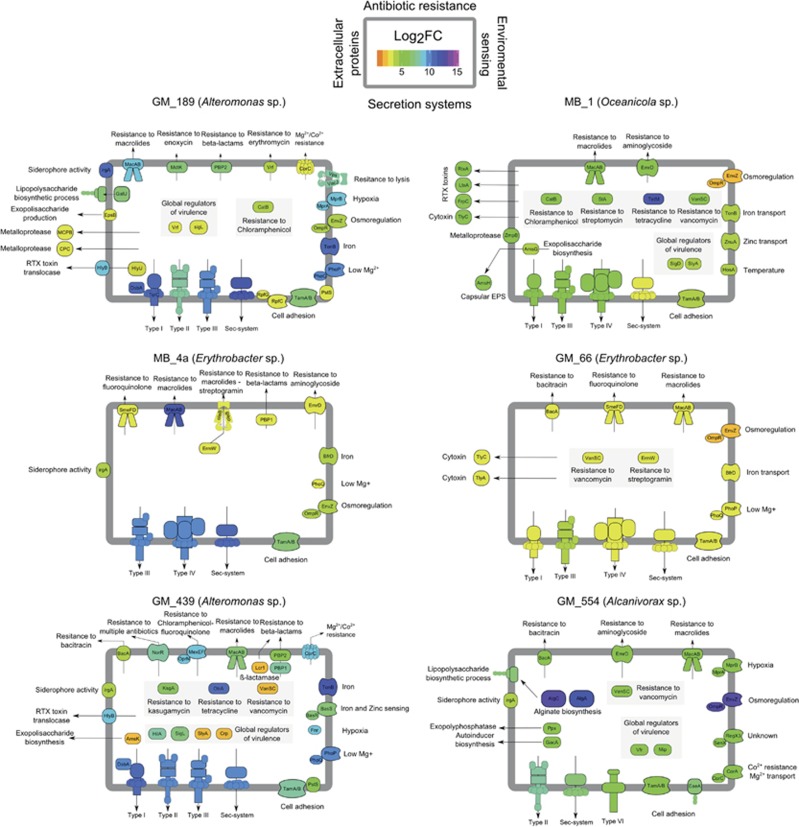
Expression of genes involved in pathogenesis in potential opportunistic pathogens (POPs). Each diagram represents a bacterial cell and each side of the cell membrane represents a group of cellular processes. Antibiotic resistance on the upper side of the membrane, environmental sensing on the right side of the membrane, bacterial secretion systems and adhesion on the lower part, and extracellular proteins and toxic compounds on the left part.

Much has been discussed regarding opportunistic pathogens in the field of coral disease and the responsible microbes, but little is known about the underlying mechanisms of disease emergence. The POPs we describe above represent candidate bacterial taxa, as they are found commonly associated with healthy organisms and ecosystems as well as disturbed ecosystems and diseased marine organisms. For instance, *Erythrobacter* and *Alteromonas* are numerously found in association with healthy marine organisms (Shigemori *et al.*, 1992; Yoon *et al.*, 2004; Koren and Rosenberg, 2006; Lampert *et al.*, 2006; Chiu *et al.*, 2007; Kalimutho *et al.*, 2007; Martínez‐García *et al.*, 2007; Vandecandelaere *et al.*, 2008; Reis *et al.*, 2009). However, their participation in coral and other marine animal diseases is strongly suggested (Cipriani *et al.*, 1980; Garland *et al.*, 1983; Pantos *et al.*, 2003; Garren *et al.*, 2008; Sunagawa *et al.*, 2009; Cárdenas *et al.*, 2012; Pollock *et al.*, 2016). In this regard, we highlight the importance of not only correlating the presence of certain bacteria with a disease, but rather looking at possible biotic and abiotic factors turning commensal bacteria into pathogens. Furthermore, the observed relative abundances of these POPs never exceeded 4% of the total community (Supplementary Table S1). This highlights the role of rare bacteria in natural communities and the need to rethink our ideas of how abundant and rare microbes contribute to ecosystem processes (Jousset *et al.*, 2017). In Figure 5, we show genes previously linked with pathogenesis in each POP. Based on each pool of virulence factors, in the following we discuss potential strategies used by the POPs to colonize and effectively outcompete native benthic bacteria on coral surfaces.

### Expression of interaction genes to promote virulence potential

A common feature among many pathogens is their ability to adhere to surfaces, colonize them, and survive adverse conditions in the form of biofilms (Dunne, 2002; Pizarro-Cerdá and Cossart, 2006). As evidence of these processes, genes involved in adhesion, such as the Type IV secretion system and the translocation and assembly module, were induced under sugar enrichment in all population genomes (Figure 5). Membrane proteins tamA and B help assemble surface structures essential to host-pathogen interactions, including adhesion and host invasion (Selkrig *et al.*, 2012), and deletion of these elements diminishes the virulence of several pathogens (Struve *et al.*, 2003; Burall *et al.*, 2004; Kelly *et al.*, 2006). Furthermore, GM_189 and GM_439 (*Alteromonas* sp.) induced *eps* and *ams* genes (Figure 5), which code for exopolysaccharide production and export proteins, that are important for biofilm formation under sugar enrichment. Biofilms not only constitute a protected lifestyle for bacteria and promote pathogenesis, but also cause impacts from an ecological perspective in coral reef ecosystems. For instance, marine biofilms containing *Alteromonas* species can influence benthic community succession by inducing the settlement and metamorphosis of cnidarian larvae and algal zoospores (Leitz, 1997; Graham *et al.*, 2000) while inhibiting the settlement of polychaetes (Dobretsov and Qian, 2002).

### Expression of genes related to iron uptake to promote virulence potential

Another group of virulence factors induced after sugar enrichment is related to iron uptake. Iron is limited in the ocean and at the same time is an essential micronutrient. Iron transporters and siderophores (iron chelating compounds) are common strategies among bacteria to acquire iron. Extracellular siderophores are rarely produced by pelagic bacteria, but are more common on particle-attached and benthic marine microorganisms (Hutchins *et al.*, 1999; Hopkinson and Barbeau, 2012). Some pathogens can produce siderophores to sequester iron from the host or other living organisms and use it to support their own growth (Amin *et al.*, 2009). The most common genes related to iron uptake processes found in the POPs were the TonB-dependent transporters and the outer protein IrgA, expressed in the population genomes GM_189 (*Alteromonas* sp.), MB_4a (*Erythrobacter*), GM_439 (*Alteromonas* sp.), and GM_554 (*Alcanivorax* sp.) (Figure 5). TonB-dependent iron acquisition is one of the main mechanisms permitting bacterial growth in a wide range of iron-limited environments, including host colonization in several pathogens (Goldberg *et al.*, 1992; Torres *et al.*, 2001; Leduc *et al.*, 2008). IrgA is the outer membrane receptor of vibriobactin, a siderophore produced by *Vibrio* species and extracellularly transported by TonB (Mey *et al.*, 2002; Kustusch *et al.*, 2011). The induction of multiple TonB elements and siderophore receptors in GM_189 and GM_439 (*Alteromonas* sp.) strongly suggests iron scavenging as an important mechanism used by these POPs for successful growth. However, little is known about marine siderophore substrates and mechanisms by which they contribute to virulence in marine pathogens.

### Expression of toxins and proteases to promote virulence potential

The third group of sugar-induced virulence factors found in the POPs was ‘toxins and proteases’. An example of a non-proteinaceous endotoxin was lipopolysaccharide, evidenced in the population genomes GM_189 (*Alteromonas* sp.) and GM_554 (*Alcanivorax* sp.) by the induction of the lipopolysaccharide biosynthetic genes *galU*, *algG*, *algA* (Figure 5). Lipopolysaccharide is involved in the modulation of marine symbiosis (Foster *et al.*, 2000), but also has an important role in the pathogenesis of several bacterial species, including *Vibrio* spp. (Nesper *et al.*, 2001), by eliciting strong immune responses in host cells. Among induced extracellular toxins, membrane-disrupting and proteolytic toxins were prevalent (Supplementary Table S4). Two extracellular zinc metalloproteases were induced by the POPs GM_189 (*Alteromonas* sp.) and MB_1 (*Oceanicola* sp.) (Figure 5). Similar extracellular zinc metalloproteases are widely distributed among several marine bacteria and largely in *Vibrio* species (Denkin and Nelson, 2004; Hasegawa *et al.*, 2008; Labreuche *et al.*, 2010). Only a few mechanisms of these metalloproteases are well described, as in the case of the hemagglutinin produced by *Vibrio cholerae* to degrade intestinal protective mucus in humans (Benitez and Silva, 2016). In addition, we provide evidence for the induction of subtilisin-like serine proteases in GM_554 (*Alcanivorax* sp.) that have antifouling activity via degradation of exopolysaccharide biofilms (Leroy *et al.*, 2008). The expression of this protease may negatively regulate the bacterium's own biofilm or compete against other bacteria. Among membrane-disrupting toxins, the cytotoxins TlyA and TlyC were induced in the POPs GM_66 (*Erythrobacter*) and MB_1 (*Oceanicola* sp.) (Figure 5). Homologs of these toxins have the ability to lyse erythrocytes, leukocytes, phagosomal vacuoles, and gastric epithelium cells (Martino *et al.*, 2001; Whitworth *et al.*, 2005). Another group of membrane-disrupting toxins induced by sugar enrichment was the calcium-dependent pore-forming cytotoxins (RTX toxins). Three RTX homologs were expressed in the POP MB_1 (*Oceanicola* sp.) and two elements involved in RTX toxin translocation were expressed in the POPs GM_189 and GM_439 (*Alteromonas* sp.) (Figure 5). RTX proteins are potent virulence factors with cytotoxic and hemolytic activities toward a broad range of animal cells (Lally *et al.*, 1999), but there is little evidence to date about the role of RTX toxins in disease. Among marine bacterial species, RTX toxins have been found in several *Vibrio* species (Kim *et al.*, 2008; Weynberg *et al.*, 2015), *Pseudoalteromonas agarivorans* (Choudhury *et al.*, 2015) and in members of the *Roseobacter* clade, including *Phaeobacter* (Durighello *et al.*, 2014) and *Ruegeria pomeroyi* (Christie-Oleza *et al.*, 2012). These proteins are characterized by tandemly repeated nonapeptides with the consensus sequence GGXGXDX[L/I/V/W/Y/F]X (where X is any amino acid) in the C-terminal half of the protein, as observed in RTX homologs expressed by MB_1 (*Oceanicola* sp.) (Supplementary Figure S5). Expression of toxin-related proteins is consistent with expression of type I to IV secretion systems that are involved in the transport of several virulence factors including effector proteins and toxins (Backert and Meyer, 2006). For instance, one main feature of all RTX toxins is their conserved mechanism of transport mediated by the type 1 secretion system (Hueck, 1998; Linhartová *et al.*, 2010). Elements of the type 1 secretion system were induced in POPs expressing RTX-related homologs, suggesting the export of these protein homologs during sugar incubations.

## Conclusions

Anthropogenic pollution and algal cover are increasingly contributing to the degradation of coral reefs worldwide by the increase of DOC concentrations. By assaying bacterial community composition and gene expression under experimental enrichment of sugars abundant in algal exudates and sewage inflows, we show that high DOC concentrations influence microbial diversity and activity, supporting a microbial lifestyle switch from commensal to pathogenic. In particular, bacteria in the genus *Alteromonas, Oceanicola, Erythrobacter*, and *Alcanivorax* provide candidate taxa for this switch, based on their copiothrophic behavior and their potential to become harmful to other marine organisms under high sugar concentrations. These POPs displayed a shift in their metabolic capabilities and expression of a wide set of virulence factors, including metalloproteases, siderophores, and toxins, as well as numerous mechanisms of antibiotic resistance. For the first time, this study shows DOC-dependent expression of VFs and shifts in metabolic capacities of coral reef microbial populations within the context of coral reef health. Although the present study has its limitations and requires validation by an *in situ* approach, our findings corroborate the DDAM and reef microbialization hypothesis, providing new mechanistic insight into the link between elevated DOC concentrations and the switch from free-living to attached stages, as well as from commensal to pathogenic lifestyles.

## Figures and Tables

**Table 1:** Coral reef bacterioplankton growth efficiencies after 48 h sugar incubations

*Treatment*	*Reef natural DOC (μmol C l^−1^)*	*DOC change (μmol C l*^−*1*^)	*Bacterial C change (μmol C l*^−*1*^)	*Bacterial specific growth rate (per day)*	*Bacterial growth efficiency*
*Exp. 1*					
Control (C1)		−14.25±5.96	1.92±0.44	0.3±0.03	0.17±0.11
Glucose (Glu)	121.55±28.23	**−362.91**±**77.5**	**11.4**±**1.72**	**0.66**±**0.03**	0.03±0.01
Galactose (Gal)		**−383.34**±**124.09**	**14.2**±**2.39**	**0.69**±**0.02**	0.04±0.01
					
*Exp. 2*					
Control (C2)		−98.08±93.2	1.82±0.65	0.45±0.15	0.03±0.01
Mannose (Man)	69.57±13.02	**−481.71**±**258.39**	1.69±0.55	0.39±0.09	**0**±**0**
Xylose (Xyl)		**−516.19**±**171.24**	2.47±0.84	0.5±0.12	**0**±**0**

Bold numbers represent significant differences (*P*<0.05) compared with the experiment control.

**Table 2:** Microbial population genomes retrieved and assembled in this study

*Population genome (experiment)*	*Completeness (%)*	*Contamination (%)*	*Bin size (Mbp)*	*GC content (%)*	*Number of PEGs*	*Suggested taxonomy*
GM_331 (E1)	98.76	2.36	3.35	62.2	3221	*Hyphomonas* sp.^a^
GM_189 (E1)	98.09	2.71	3.3	45.4	3207	*Alteromonas* sp.^a^
GM_23 (E1)	97.66	0	2.92	55.1	2982	Rhodobacteraceae
MB_1 (E2)	95.12	1.8	3.59	63	3408	*Oceanicola* sp.
MB_3 (E1)	92.2	0.56	2.07	63.1	1747	Flavobacteraceae
GM_439 (E1)	90.79	3.35	4.72	45.8	3736	*Alteromonas* sp.
MB_4a (E1)	90.69	1.64	2.85	64.3	2724	*Erythrobacter* sp.
GM_293 (E2)	88.2	5	2.15	53.8	2094	Alphaproteobacteria
GM_554 (E2)	87.65	5.44	2.76	59.8	2451	*Alcanivorax* sp.^a^
merged_357_358 (E1)	83.36	8.21	2.8	66.6	2619	Actinobacteria
GM_66 (E2)	81.61	6.68	2.37	64.3	2257	*Erythrobacter* sp
GM_20 (E1)	81.4	1.91	2.64	43.82	2283	*Pseudoalteromonas*
GM_34 (E2)	80.4	3.2	1.54	55.6	1379	Euryarchaeota
merged_809_810 (E1)	80.36	8.27	3.38	48.7	2993	*Alteromonas* sp
MB_2 (E2)	80.24	0	1.46	47.3	1292	Flavobacteraceae
MB_5a (E1)	79.34	2.42	1.68	59.3	1625	Alphaproteobacteria
GM_808 (E1)	77.7	2.83	5.17	59.8	4654	*Labrenzia sp.*
MB_4b (E2)	76.2	0.01	1.76	58.8	1706	Alphaproteobacteria
GM_44 (E2)	76.12	1.95	3.57	59.5	3388	*Ruegeria mobilis*
MB_5b (E2)	75.63	1.54	1.7	57.6	1463	Flavobacteraceae
GM_50 (E2)	73.74	7.13	3.06	45.11	2625	*Alteromonas macleodii*
GM_88 (E2)	73.42	4.5	2.34	44.5	2112	Gammaproteobacteria
MB_7 (E1)	72.08	0.43	1.31	57.4	1358	Actinobacteria
merged_515_516 (E1)	72.08	5.22	2.25	61.5	2018	Rhodobacteraceae
merged_287_630 (E2)	71.62	8.61	2.03	63	1907	Alphaproteobacteria
MB_8 (E1)	71.33	0	1.09	50.3	998	Euryarchaeota
MB_9 (E1)	71.2	0.27	1.61	60.6	1419	Euryarchaeota

Abbreviation: PEGs, protein-encoding genes.

a Population genome contains 16 S rRNA gene.

## Supplementary Material

Supplementray Information

## References

[bib1] Al-Dahash LM, Mahmoud HM. (2013). Harboring oil-degrading bacteria: a potential mechanism of adaptation and survival in corals inhabiting oil-contaminated reefs. Marine Poll Bull 72: 364–374. 10.1016/j.marpolbul.2012.08.029 23014479

[bib2] Alker AP, Smith GW, Kim K. (2001). Characterization of Aspergillus sydowii (Thom et Church), a fungal pathogen of Caribbean sea fan corals. Hydrobiologia 460: 105–111.

[bib3] Amin SA, Green DH, Küpper FC, Carrano CJ. (2009). Vibrioferrin, an unusual marine siderophore: iron binding, photochemistry, and biological implications. Inorganic Chem 48: 11451–11458. 10.1021/ic9016883 19821595

[bib4] Andersson AF, Lindberg M, Jakobsson H, Bäckhed F, Nyrén P, Engstrand L. (2008). Comparative analysis of human gut microbiota by barcoded pyrosequencing. PloS One 3: e2836. 18665274 10.1371/journal.pone.0002836 PMC2475661

[bib5] Aziz RK, Bartels D, Best AA, DeJongh M, Disz T, Edwards RA et al. (2008). The RAST Server: rapid annotations using subsystems technology. BMC Genomics 9: 1. 18261238 10.1186/1471-2164-9-75 PMC2265698

[bib6] Backert S, Meyer TF. (2006). Type IV secretion systems and their effectors in bacterial pathogenesis. Curr Opin Microbiol 9: 207–217. 16529981 10.1016/j.mib.2006.02.008

[bib7] Baker AC, Glynn PW, Riegl B. (2008). Climate change and coral reef bleaching: an ecological assessment of long-term impacts, recovery trends and future outlook. Estuar Coast Shelf Sci 80: 435–471.

[bib8] Banin E, Vassilakos D, Orr E, Martinez RJ, Rosenberg E. (2003). Superoxide dismutase is a virulence factor produced by the coral bleaching pathogen Vibrio shiloi. Curr Microbiol 46: 0418–0422. 10.1007/s00284-002-3912-5 12732948

[bib9] Barott KL, Rohwer FL. (2012). Unseen players shape benthic competition on coral reefs. Trends Microbiol 20: 621–628. 22944243 10.1016/j.tim.2012.08.004

[bib10] Bayer T, Neave MJ, Alsheikh-Hussain A, Aranda M, Yum LK, Mincer T et al. (2013). The microbiome of the Red Sea coral Stylophora pistillata is dominated by tissue-associated Endozoicomonas bacteria. Appl Environ Microbiol 79: 4759–4762. 23709513 10.1128/AEM.00695-13 PMC3719505

[bib11] Benitez JA, Silva AJ. (2016). Vibrio cholerae hemagglutinin (HA)/protease: an extracellular metalloprotease with multiple pathogenic activities. Toxicon 115: 55–62. 26952544 10.1016/j.toxicon.2016.03.003 PMC4828278

[bib12] Blencke H-M, Homuth G, Ludwig H, Mäder U, Hecker M, Stülke J. (2003). Transcriptional profiling of gene expression in response to glucose in Bacillus subtilis: regulation of the central metabolic pathways. Metab Eng 5: 133–149. 12850135 10.1016/s1096-7176(03)00009-0

[bib13] Bolger AM, Lohse M, Usadel B. (2014). Trimmomatic: a flexible trimmer for Illumina sequence data. Bioinformatics 30: 2114–2120. 24695404 10.1093/bioinformatics/btu170 PMC4103590

[bib14] Bourne DG, Munn CB. (2005). Diversity of bacteria associated with the coral Pocillopora damicornis from the Great Barrier Reef. Environ Microbiol 7: 1162–1174. 16011753 10.1111/j.1462-2920.2005.00793.x

[bib15] Brown SP, Cornforth DM, Mideo N. (2012). Evolution of virulence in opportunistic pathogens: generalism, plasticity, and control. Trends Microbiol 20: 336–342. 22564248 10.1016/j.tim.2012.04.005 PMC3491314

[bib16] Brückner R, Titgemeyer F. (2002). Carbon catabolite repression in bacteria: choice of the carbon source and autoregulatory limitation of sugar utilization. FEMS Microbiol Lett 209: 141–148. 12007797 10.1111/j.1574-6968.2002.tb11123.x

[bib17] Bruno JF, Petes LE, Drew Harvell C, Hettinger A. (2003). Nutrient enrichment can increase the severity of coral diseases. Ecol Lett 6: 1056–1061.

[bib18] Burall LS, Harro JM, Li X, Lockatell CV, Himpsl SD, Hebel JR et al. (2004). Proteus mirabilis genes that contribute to pathogenesis of urinary tract infection: identification of 25 signature-tagged mutants attenuated at least 100-fold. Infect Immun 72: 2922–2938. 15102805 10.1128/IAI.72.5.2922-2938.2004 PMC387873

[bib19] Cantarel BL, Coutinho PM, Rancurel C, Bernard T, Lombard V, Henrissat B. (2009). The Carbohydrate-Active EnZymes database (CAZy): an expert resource for glycogenomics. Nucleic Acids Res 37: D233–D238. 18838391 10.1093/nar/gkn663 PMC2686590

[bib20] Cárdenas A, Rodriguez-R LM, Pizarro V, Cadavid LF, Arévalo-Ferro C. (2012). Shifts in bacterial communities of two Caribbean reef-building coral species affected by white plague disease. ISME J 6: 502–512. 21955993 10.1038/ismej.2011.123 PMC3280130

[bib21] Chen L, Yang J, Yu J, Yao Z, Sun L, Shen Y et al. (2005). VFDB: a reference database for bacterial virulence factors. Nucleic Acids Res 33: D325–D328. 15608208 10.1093/nar/gki008 PMC539962

[bib22] Chimetto LA, Brocchi M, Thompson CC, Martins RC, Ramos HR, Thompson FL. (2008). Vibrios dominate as culturable nitrogen-fixing bacteria of the Brazilian coral Mussismilia hispida. Syst Appl Microbiol 31: 312–319. 18678453 10.1016/j.syapm.2008.06.001

[bib23] Chiu H-H, Shieh WY, Lin SY, Tseng C-M, Chiang P-W, Wagner-Döbler I. (2007). Alteromonas tagae sp. nov. and Alteromonas simiduii sp. nov., mercury-resistant bacteria isolated from a Taiwanese estuary. Int J Syst Evol Microbiol 57: 1209–1216. 17551031 10.1099/ijs.0.64762-0

[bib24] Choudhury JD, Pramanik A, Webster NS, Llewellyn LE, Gachhui R, Mukherjee J. (2015). The pathogen of the Great Barrier Reef sponge Rhopaloeides odorabile is a new strain of Pseudoalteromonas agarivorans containing abundant and diverse virulence-related genes. Mar Biotechnol 17: 463–478. 25837832 10.1007/s10126-015-9627-y

[bib25] Christie-Oleza JA, Piña-Villalonga JM, Bosch R, Nogales B, Armengaud J. (2012). Comparative proteogenomics of twelve Roseobacter exoproteomes reveals different adaptive strategies among these marine bacteria. Mol Cell Proteomics 11: M111. 013110. 10.1074/mcp.M111.013110 PMC3277765 22122883

[bib26] Cipriani GR, Wheeler RS, Sizemore RK. (1980). Characterization of brown spot disease of Gulf Coast shrimp. J Invertebr Pathol 36: 255–263.

[bib27] Cottrell MT, Kirchman DL. (2016). Transcriptional control in marine copiotrophic and oligotrophic bacteria with streamlined genomes. Appl Environ Microbiol 82: 6010–6018. 27474718 10.1128/AEM.01299-16 PMC5038029

[bib28] Cramer KL, Jackson JB, Angioletti CV, Leonard‐Pingel J, Guilderson TP. (2012). Anthropogenic mortality on coral reefs in Caribbean Panama predates coral disease and bleaching. Ecol Lett 15: 561–567. 22462739 10.1111/j.1461-0248.2012.01768.x

[bib29] Dale C, Moran NA. (2006). Molecular interactions between bacterial symbionts and their hosts. Cell 126: 453–465. 16901780 10.1016/j.cell.2006.07.014

[bib30] Daniels C, Baumgarten S, Yum LK, Michell CT, Bayer T, Arif C et al. (2015). Metatranscriptome analysis of the reef-building coral Orbicella faveolata indicates holobiont response to coral disease. Front Mar Sci 2: 62.

[bib31] Denkin SM, Nelson DR. (2004). Regulation of Vibrio anguillarum empA metalloprotease expression and its role in virulence. Appl Environ Microbiol 70: 4193–4204. 15240301 10.1128/AEM.70.7.4193-4204.2004 PMC444792

[bib32] Deutscher J, Francke C, Postma PW. (2006). How phosphotransferase system-related protein phosphorylation regulates carbohydrate metabolism in bacteria. Microbiol Mol Biol Rev 70: 939–1031. 17158705 10.1128/MMBR.00024-06 PMC1698508

[bib33] Dobretsov SV, Qian P-Y. (2002). Effect of bacteria associated with the green alga Ulva reticulata on marine micro-and macrofouling. Biofouling 18: 217–228.

[bib34] Dunne WM. (2002). Bacterial adhesion: seen any good biofilms lately? Clin Microbiol Rev 15: 155–166. 11932228 10.1128/CMR.15.2.155-166.2002 PMC118072

[bib35] Durighello E, Christie-Oleza JA, Armengaud J. (2014). Assessing the exoproteome of marine bacteria, lesson from a RTX-toxin abundantly secreted by Phaeobacter strain DSM 17395. PloS One 9: e89691. 24586966 10.1371/journal.pone.0089691 PMC3933643

[bib36] Edgar RC, Haas BJ, Clemente JC, Quince C, Knight R. (2011). UCHIME improves sensitivity and speed of chimera detection. Bioinformatics 27: 2194–2200. 21700674 10.1093/bioinformatics/btr381 PMC3150044

[bib37] Eiler A, Langenheder S, Bertilsson S, Tranvik LJ. (2003). Heterotrophic bacterial growth efficiency and community structure at different natural organic carbon concentrations. Appl Environ Microbiol 69: 3701–3709. 12839735 10.1128/AEM.69.7.3701-3709.2003 PMC165184

[bib38] Flamholz A, Noor E, Bar-Even A, Liebermeister W, Milo R. (2013). Glycolytic strategy as a tradeoff between energy yield and protein cost. Proc Natl Acad Sci USA 110: 10039–10044. 23630264 10.1073/pnas.1215283110 PMC3683749

[bib39] Foster J, Apicella M, McFall-Ngai M. (2000). Vibrio fischeri lipopolysaccharide induces developmental apoptosis, but not complete morphogenesis, of the Euprymna scolopes symbiotic light organ. Dev Biol 226: 242–254. 11023684 10.1006/dbio.2000.9868

[bib40] Frias-Lopez J, Shi Y, Tyson GW, Coleman ML, Schuster SC, Chisholm SW et al. (2008). Microbial community gene expression in ocean surface waters. Proc Natl Acad Sci U.S.A. 105: 3805–3810. 18316740 10.1073/pnas.0708897105 PMC2268829

[bib41] Frias-Lopez J, Zerkle AL, Bonheyo GT, Fouke BW. (2002). Partitioning of bacterial communities between seawater and healthy, black band diseased, and dead coral surfaces. Appl Environ Microbiol 68: 2214–2228. 11976091 10.1128/AEM.68.5.2214-2228.2002 PMC127591

[bib42] Fukuda R, Ogawa H, Nagata T, Koike I. (1998). Direct determination of carbon and nitrogen contents of natural bacterial assemblages in marine environments. Appl Environ Microbiol 64: 3352–3358. 9726882 10.1128/aem.64.9.3352-3358.1998 PMC106732

[bib43] Garland C, Nash G, Summer C, McMeekin T. (1983). Bacterial pathogens of oyster larvae (Crassostrea gigas) in a Tasmanian hatchery. Mar Freshwater Res 34: 483–487.

[bib44] Garren M, Smriga S, Azam F. (2008). Gradients of coastal fish farm effluents and their effect on coral reef microbes. Environ Microbiol 10: 2299–2312. 18557772 10.1111/j.1462-2920.2008.01654.x

[bib45] Goldberg MB, Boyko SA, Butterton JR, Stoebner JA, Payne SM, Calderwood SB. (1992). Characterization of a Vibrio cholerae virulence factor homologous to the family of TonB‐dependent proteins. Mol Microbiol 6: 2407–2418. 1406279 10.1111/j.1365-2958.1992.tb01415.x

[bib46] Görke B, Stülke J. (2008). Carbon catabolite repression in bacteria: many ways to make the most out of nutrients. Nature Rev Microbiol 6: 613–624. 18628769 10.1038/nrmicro1932

[bib47] Graham PD, Joint I, Nevell TG, Smith JR, Stone M, Tsibouklis J. (2000). Bacterial colonisation and settlement of algal spores and barnacle larvae on low surface energy materials. Biofouling 16: 289–299.

[bib48] Haas AF, Fairoz MF, Kelly LW, Nelson CE, Dinsdale EA, Edwards RA et al. (2016). Global microbialization of coral reefs. Nat Microbiol 1: 16042. 27572833 10.1038/nmicrobiol.2016.42

[bib49] Haas AF, Nelson CE, Kelly LW, Carlson CA, Rohwer F, Leichter JJ et al. (2011). Effects of coral reef benthic primary producers on dissolved organic carbon and microbial activity. PloS ONE 6: e27973. 22125645 10.1371/journal.pone.0027973 PMC3220721

[bib50] Haas AF, Nelson CE, Rohwer F, Wegley-Kelly L, Quistad SD, Carlson CA et al. (2013). Influence of coral and algal exudates on microbially mediated reef metabolism. PeerJ 1: e108. 23882445 10.7717/peerj.108 PMC3719129

[bib51] Haas AF, Wild C. (2010). Composition analysis of organic matter released by cosmopolitan coral reef-associated green algae. Aquatic Biol 10: 131–138.

[bib52] Harvell C, Kim K, Burkholder J, Colwell R, Epstein PR, Grimes D et al. (1999). Emerging marine diseases—climate links and anthropogenic factors. Science 285: 1505–1510. 10498537 10.1126/science.285.5433.1505

[bib53] Hasegawa H, Lind EJ, Boin MA, Häse CC. (2008). The extracellular metalloprotease of Vibrio tubiashii is a major virulence factor for pacific oyster (Crassostrea gigas) larvae. Appl Environ Microbiol 74: 4101–4110. 18456850 10.1128/AEM.00061-08 PMC2446533

[bib54] Henderson B, Martin A. (2011). Bacterial virulence in the moonlight: multitasking bacterial moonlighting proteins are virulence determinants in infectious disease. Infect Immun 79: 3476–3491. 21646455 10.1128/IAI.00179-11 PMC3165470

[bib55] Hillman K, Goodrich-Blair H. (2016). Are you my symbiont? Microbial polymorphic toxins and antimicrobial compounds as honest signals of beneficial symbiotic defensive traits. Curr Opin Microbiol 31: 184–190. 27128187 10.1016/j.mib.2016.04.010

[bib56] Hoegh-Guldberg O, Mumby PJ, Hooten AJ, Steneck RS, Greenfield P, Gomez E et al. (2007). Coral reefs under rapid climate change and ocean acidification. Science 318: 1737–1742. 18079392 10.1126/science.1152509

[bib57] Holmfeldt K, Middelboe M, Nybroe O, Riemann L. (2007). Large variabilities in host strain susceptibility and phage host range govern interactions between lytic marine phages and their Flavobacterium hosts. Appl Environ Microbiol 73: 6730–6739. 17766444 10.1128/AEM.01399-07 PMC2074958

[bib58] Hopkinson BM, Barbeau KA. (2012). Iron transporters in marine prokaryotic genomes and metagenomes. Environ Microbiol 14: 114–128. 21883791 10.1111/j.1462-2920.2011.02539.x

[bib59] Huang M-h, Li Y-m, Gu G-w. (2010). Chemical composition of organic matters in domestic wastewater. Desalination 262: 36–42.

[bib60] Hueck CJ. (1998). Type III protein secretion systems in bacterial pathogens of animals and plants. Microbiol Mol Biol Rev 62: 379–433. 9618447 10.1128/mmbr.62.2.379-433.1998 PMC98920

[bib61] Hughes TP, Baird AH, Bellwood DR, Card M, Connolly SR, Folke C et al. (2003). Climate change, human impacts, and the resilience of coral reefs. Science 301: 929–933. 12920289 10.1126/science.1085046

[bib62] Hutchins DA, Witter AE, Butler A, Luther GW. (1999). Competition among marine phytoplankton for different chelated iron species. Nature 400: 858–861.

[bib63] Hyatt D, Chen G-L, LoCascio PF, Land ML, Larimer FW, Hauser LJ. (2010). Prodigal: prokaryotic gene recognition and translation initiation site identification. BMC Bioinformatics 11: 1. 20211023 10.1186/1471-2105-11-119 PMC2848648

[bib64] Imelfort M, Parks D, Woodcroft BJ, Dennis P, Hugenholtz P, Tyson GW. (2014). GroopM: an automated tool for the recovery of population genomes from related metagenomes. PeerJ 2: e603. 25289188 10.7717/peerj.603 PMC4183954

[bib65] Jackson JB, Kirby MX, Berger WH, Bjorndal KA, Botsford LW, Bourque BJ et al. (2001). Historical overfishing and the recent collapse of coastal ecosystems. Science 293: 629–637. 11474098 10.1126/science.1059199

[bib66] Jahreis K, Pimentel-Schmitt EF, Brückner R, Titgemeyer F. (2008). Ins and outs of glucose transport systems in eubacteria. FEMS Microbiol Rev 32: 891–907. 18647176 10.1111/j.1574-6976.2008.00125.x

[bib67] Jousset A, Bienhold C, Chatzinotas A, Gallien L, Gobet A, Kurm V et al. (2017). Where less may be more: how the rare biosphere pulls ecosystems strings. ISME J 11: 853–862. 28072420 10.1038/ismej.2016.174 PMC5364357

[bib68] Kalimutho M, Ahmad A, Kassim Z. (2007). Isolation, Characterization and Identification of Bacteria associated with Mucus of Acropora cervicornis Coral from Bidong Island, Terengganu, Malaysia. J Sci 26: 27–39.

[bib69] Kang DD, Froula J, Egan R, Wang Z. (2015). MetaBAT, an efficient tool for accurately reconstructing single genomes from complex microbial communities. PeerJ 3: e1165. 26336640 10.7717/peerj.1165 PMC4556158

[bib70] Kelly M, Hart E, Mundy R, Marches O, Wiles S, Badea L et al. (2006). Essential role of the type III secretion system effector NleB in colonization of mice by Citrobacter rodentium. Infect Immun 74: 2328–2337. 16552063 10.1128/IAI.74.4.2328-2337.2006 PMC1418941

[bib71] Kennedy J, Baker P, Piper C, Cotter PD, Walsh M, Mooij MJ et al. (2009). Isolation and analysis of bacteria with antimicrobial activities from the marine sponge Haliclona simulans collected from Irish waters. Mar Biotechnol 11: 384–396. 18953608 10.1007/s10126-008-9154-1

[bib72] Kim YR, Lee SE, Kook H, Yeom JA, Na HS, Kim SY et al. (2008). Vibrio vulnificus RTX toxin kills host cells only after contact of the bacteria with host cells. Cell Microbiol 10: 848–862. 18005241 10.1111/j.1462-5822.2007.01088.x

[bib73] Kimes NE, Grim CJ, Johnson WR, Hasan NA, Tall BD, Kothary MH et al. (2012). Temperature regulation of virulence factors in the pathogen Vibrio coralliilyticus. ISME J 6: 835–846. 22158392 10.1038/ismej.2011.154 PMC3309362

[bib74] Kline DI, Kuntz NM, Breitbart M, Knowlton N, Rohwer F. (2006). Role of elevated organic carbon levels and microbial activity in coral mortality. Mar Ecol Prog Ser 314: 119–125.

[bib75] Kopylova E, Noé L, Touzet H. (2012). SortMeRNA: fast and accurate filtering of ribosomal RNAs in metatranscriptomic data. Bioinformatics 28: 3211–3217. 23071270 10.1093/bioinformatics/bts611

[bib76] Koren O, Rosenberg E. (2006). Bacteria associated with mucus and tissues of the coral Oculina patagonica in summer and winter. Appl Environ Microbiol 72: 5254–5259. 16885273 10.1128/AEM.00554-06 PMC1538755

[bib77] Kuntz NM, Kline DI, Sandin SA, Rohwer F. (2005). Pathologies and mortality rates caused by organic carbon and nutrient stressors in three Caribbean coral species. Mar Ecol Prog Ser 294: 173–180.

[bib78] Kustusch RJ, Kuehl CJ, Crosa JH. (2011). Power plays: iron transport and energy transduction in pathogenic vibrios. BioMetals 24: 559–566. 21399938 10.1007/s10534-011-9437-2 PMC3092006

[bib79] Labreuche Y, Le Roux F, Henry J, Zatylny C, Huvet A, Lambert C et al. (2010). Vibrio aestuarianus zinc metalloprotease causes lethality in the Pacific oyster Crassostrea gigas and impairs the host cellular immune defenses. Fish Shellfish Immunol 29: 753–758. 20624467 10.1016/j.fsi.2010.07.007

[bib80] Lally ET, Hill RB, Kieba IR, Korostoff J. (1999). The interaction between RTX toxins and target cells. Trends Microbiol 7: 356–361. 10470043 10.1016/s0966-842x(99)01530-9

[bib81] Lampert Y, Kelman D, Dubinsky Z, Nitzan Y, Hill RT. (2006). Diversity of culturable bacteria in the mucus of the Red Sea coral Fungia scutaria. FEMS Microbiol Ecol 58: 99–108. 16958911 10.1111/j.1574-6941.2006.00136.x

[bib82] Landsberg JH. (1995). Tropical reef-fish disease outbreaks and mass mortalities in Florida, USA: what is the role of dietary biological toxins? Dis Aquat Organ 22: 83–100.

[bib83] Langmead B, Salzberg SL. (2012). Fast gapped-read alignment with Bowtie 2. Nature Med 9: 357–359. 10.1038/nmeth.1923 PMC3322381 22388286

[bib84] Lauro FM, McDougald D, Thomas T, Williams TJ, Egan S, Rice S et al. (2009). The genomic basis of trophic strategy in marine bacteria. Proc Natl Acad Sci USA 106: 15527–15533. 19805210 10.1073/pnas.0903507106 PMC2739866

[bib85] Leduc I, Banks KE, Fortney KR, Patterson KB, Billings SD, Katz BP et al. (2008). Evaluation of the repertoire of the TonB-dependent receptors of Haemophilus ducreyi for their role in virulence in humans. J Infect Dis 197: 1103–1109. 18462159 10.1086/586901

[bib86] Lee ST, Davy SK, Tang S-L, Kench PS. (2016). Mucus sugar content shapes the bacterial community structure in thermally stressed acropora muricata. Front Microbiol 7: 371. 27047481 10.3389/fmicb.2016.00371 PMC4805648

[bib87] Leitz T. (1997). Induction of settlement and metamorphosis of cnidarian larvae: signals and signal transduction. Int J Invertebr Repr Dev 31: 109–122.

[bib88] Leroy C, Delbarre C, Ghillebaert F, Compere C, Combes D. (2008). Influence of subtilisin on the adhesion of a marine bacterium which produces mainly proteins as extracellular polymers. J Appl Microbiol 105: 791–799. 18489561 10.1111/j.1365-2672.2008.03837.x

[bib89] Li D, Liu C-M, Luo R, Sadakane K, Lam T-W. (2015). MEGAHIT: an ultra-fast single-node solution for large and complex metagenomics assembly via succinct de Bruijn graph. Bioinformatics 31: 1674–1676. 25609793 10.1093/bioinformatics/btv033

[bib90] Linhartová I, Bumba L, Mašín J, Basler M, Osička R, Kamanová J et al. (2010). RTX proteins: a highly diverse family secreted by a common mechanism. FEMS Microbiol Rev 34: 1076–1112. 20528947 10.1111/j.1574-6976.2010.00231.x PMC3034196

[bib91] Liu B, Pop M. (2009). ARDB—antibiotic resistance genes database. Nucleic Acids Res 37: D443–D447. 18832362 10.1093/nar/gkn656 PMC2686595

[bib92] Long RA, Azam F. (2001). Antagonistic interactions among marine pelagic bacteria. Appl Environ Microbiol 67: 4975–4983. 11679315 10.1128/AEM.67.11.4975-4983.2001 PMC93260

[bib93] Martínez‐García M, Díaz‐Valdés M, Wanner G, Ramos‐Esplá A, Antón J. (2007). Microbial community associated with the colonial ascidian Cystodytes dellechiajei. Environ Microbiol 9: 521–534. 17222150 10.1111/j.1462-2920.2006.01170.x

[bib94] Martino MC, Stabler RA, Zhang ZW, Farthing MJ, Wren BW, Dorrell N. (2001). Helicobacter pylori pore-forming cytolysin orthologue TlyA possesses *in vitro* hemolytic activity and has a role in colonization of the gastric mucosa. Infect Immun 69: 1697–1703. 11179345 10.1128/IAI.69.3.1697-1703.2001 PMC98074

[bib95] McCook L. (1999). Macroalgae, nutrients and phase shifts on coral reefs: scientific issues and management consequences for the Great Barrier Reef. Coral Reefs 18: 357–367.

[bib96] McDole T, Nulton J, Barott KL, Felts B, Hand C, Hatay M et al. (2012). Assessing coral reefs on a Pacific-wide scale using the microbialization score. PloS One 7: e43233. 22970122 10.1371/journal.pone.0043233 PMC3436891

[bib97] McDonald D, Price MN, Goodrich J, Nawrocki EP, DeSantis TZ, Probst A et al. (2012). An improved Greengenes taxonomy with explicit ranks for ecological and evolutionary analyses of bacteria and archaea. ISME J 6: 610–618. 22134646 10.1038/ismej.2011.139 PMC3280142

[bib98] McMurdie PJ, Holmes S. (2013). phyloseq: an R package for reproducible interactive analysis and graphics of microbiome census data. PloS One 8: e61217. 23630581 10.1371/journal.pone.0061217 PMC3632530

[bib99] Mende DR, Sunagawa S, Zeller G, Bork P. (2013). Accurate and universal delineation of prokaryotic species. Nature Med 10: 881–884. 10.1038/nmeth.2575 23892899

[bib100] Mey AR, Wyckoff EE, Oglesby AG, Rab E, Taylor RK, Payne SM. (2002). Identification of the Vibrio cholerae enterobactin receptors VctA and IrgA: IrgA is not required for virulence. Infect Immun 70: 3419–3426. 12065481 10.1128/IAI.70.7.3419-3426.2002 PMC128051

[bib101] Middelboe M, Hagström A, Blackburn N, Sinn B, Fischer U, Borch N et al. (2001). Effects of bacteriophages on the population dynamics of four strains of pelagic marine bacteria. Microb Ecol 42: 395–406. 12024264 10.1007/s00248-001-0012-1

[bib102] Mohamed NM, Colman AS, Tal Y, Hill RT. (2008). Diversity and expression of nitrogen fixation genes in bacterial symbionts of marine sponges. Environ Microbiol 10: 2910–2921. 18761667 10.1111/j.1462-2920.2008.01704.x

[bib103] Muller EM, Woesik R. (2012). Caribbean coral diseases: primary transmission or secondary infection? Glob Chang Biol 18: 3529–3535.

[bib104] Nair S, Simidu U. (1987). Distribution and significance of heterotrophic marine bacteria with antibacterial activity. Appl Environ Microbiol 53: 2957–2962. 3435149 10.1128/aem.53.12.2957-2962.1987 PMC204229

[bib105] Nelson CE, Alldredge AL, McCliment EA, Amaral-Zettler LA, Carlson CA. (2011). Depleted dissolved organic carbon and distinct bacterial communities in the water column of a rapid-flushing coral reef ecosystem. ISME J 5: 1374–1387. 21390080 10.1038/ismej.2011.12 PMC3146267

[bib106] Nelson CE, Goldberg SJ, Kelly LW, Haas AF, Smith JE, Rohwer F et al. (2013). Coral and macroalgal exudates vary in neutral sugar composition and differentially enrich reef bacterioplankton lineages. ISME J 7: 962–979. 23303369 10.1038/ismej.2012.161 PMC3635233

[bib107] Nesper J, Lauriano CM, Klose KE, Kapfhammer D, Kraiß A, Reidl J. (2001). Characterization of Vibrio cholerae O1 El TorgalU and galE Mutants: Influence on lipopolysaccharide structure, colonization, and biofilm formation. Infect Immun 69: 435–445. 11119535 10.1128/IAI.69.1.435-445.2001 PMC97901

[bib108] Nissimov J, Rosenberg E, Munn CB. (2009). Antimicrobial properties of resident coral mucus bacteria of Oculina patagonica. FEMS Microbiol Lett 292: 210–215. 19191871 10.1111/j.1574-6968.2009.01490.x

[bib109] Overbeek R, Olson R, Pusch GD, Olsen GJ, Davis JJ, Disz T et al. (2014). The SEED and the rapid annotation of microbial genomes using Subsystems Technology (RAST). Nucleic Acids Res 42: D206–D214. 24293654 10.1093/nar/gkt1226 PMC3965101

[bib110] Pantos O, Cooney RP, Le Tissier MD, Barer MR, O'Donnell AG, Bythell JC. (2003). The bacterial ecology of a plague‐like disease affecting the Caribbean coral Montastrea annularis. Environ Microbiol 5: 370–382. 12713463 10.1046/j.1462-2920.2003.00427.x

[bib111] Parks DH, Imelfort M, Skennerton CT, Hugenholtz P, Tyson GW. (2015). CheckM: assessing the quality of microbial genomes recovered from isolates, single cells, and metagenomes. Genome Res 25: 1043–1055. 25977477 10.1101/gr.186072.114 PMC4484387

[bib112] Patil KR, Roune L, McHardy AC. (2012). The PhyloPythiaS web server for taxonomic assignment of metagenome sequences. PloS One 7: e38581. 22745671 10.1371/journal.pone.0038581 PMC3380018

[bib113] Pizarro-Cerdá J, Cossart P. (2006). Bacterial adhesion and entry into host cells. Cell 124: 715–727. 16497583 10.1016/j.cell.2006.02.012

[bib114] Pollock FJ, Wada N, Torda G, Willis BL, Bourne DG. (2016). Repeated sampling of white syndrome-affected corals reveals distinct microbiome at disease lesion fronts. Appl Environ Microbiol, 02799–02716. 10.1128/AEM.02799-16 PMC5203618 27815275

[bib115] Porter JW, Dustan P, Jaap WC, Patterson KL, Kosmynin V, Meier OW et al. (2001). Patterns of spread of coral disease in the Florida Keys In The Ecology and Etiology of Newly Emerging Marine Diseases. Springer, Netherlands, pp 1–24.

[bib116] Postma P, Lengeler J, Jacobson G. (1993). Phosphoenolpyruvate: carbohydrate phosphotransferase systems of bacteria. Microbiol Rev 57: 543–594. 8246840 10.1128/mr.57.3.543-594.1993 PMC372926

[bib117] Quast C, Pruesse E, Yilmaz P, Gerken J, Schweer T, Yarza P et al. (2013). The SILVA ribosomal RNA gene database project: improved data processing and web-based tools. Nucleic Acids Res 41: D590–D596. 23193283 10.1093/nar/gks1219 PMC3531112

[bib118] Reis A, Araújo Jr S, Moura R, Francini‐Filho R, Pappas JrG, Coelho A et al. (2009). Bacterial diversity associated with the Brazilian endemic reef coral Mussismilia braziliensis. J Appl Microbiol 106: 1378–1387. 19187136 10.1111/j.1365-2672.2008.04106.x

[bib119] Richardson LL, Goldberg WM, Kuta KG, Aronson RB, Smith GW, Ritchie KB et al. (1998). Florida's mystery coral-killer identified. Nature 392: 557–558.

[bib120] Richter M, Rosselló-Móra R. (2009). Shifting the genomic gold standard for the prokaryotic species definition. Proc Natl Acad Sci USA 106: 19126–19131. 19855009 10.1073/pnas.0906412106 PMC2776425

[bib121] Ritchie KB. (2006). Regulation of microbial populations by coral surface mucus and mucus-associated bacteria. Mar Ecol Prog Ser 322: 1–14.

[bib122] Roberts A, Pachter L. (2013). Streaming fragment assignment for real-time analysis of sequencing experiments. Nat Med 10: 71–73. 10.1038/nmeth.2251 PMC3880119 23160280

[bib123] Roder C, Arif C, Daniels C, Weil E, Voolstra CR. (2014). Bacterial profiling of White Plague Disease across corals and oceans indicates a conserved and distinct disease microbiome. Mol Ecol 23: 965–974. 24350609 10.1111/mec.12638 PMC4285310

[bib124] Rodriguez-R LM, Konstantinidis KT. (2016) The enveomics collection: a toolbox for specialized analyses of microbial genomes and metagenomes (2167-9843).

[bib125] Roik A, Röthig T, Roder C, Ziegler M, Kremb S, Voolstra C. (2016). Year-long monitoring of physico-chemical and biological variables provide a comparative baseline of coral reef functioning in the Central Red Sea. PloS One 11: e0163939. 27828965 10.1371/journal.pone.0163939 PMC5102394

[bib126] Rosenberg E, Koren O, Reshef L, Efrony R, Zilber-Rosenberg I. (2007). The role of microorganisms in coral health, disease and evolution. Nature Rev Microbiol 5: 355–362. 17384666 10.1038/nrmicro1635

[bib127] Schloss PD, Westcott SL, Ryabin T, Hall JR, Hartmann M, Hollister EB et al. (2009). Introducing mothur: open-source, platform-independent, community-supported software for describing and comparing microbial communities. Appl Environ Microbiol 75: 7537–7541. 19801464 10.1128/AEM.01541-09 PMC2786419

[bib128] Selkrig J, Mosbahi K, Webb CT, Belousoff MJ, Perry AJ, Wells TJ et al. (2012). Discovery of an archetypal protein transport system in bacterial outer membranes. Nat Struct Mol Biol 19: 506–510. 22466966 10.1038/nsmb.2261

[bib129] Shi Y, Tyson GW, Eppley JM, DeLong EF. (2011). Integrated metatranscriptomic and metagenomic analyses of stratified microbial assemblages in the open ocean. ISME J 5: 999–1013. 21151004 10.1038/ismej.2010.189 PMC3131857

[bib130] Shigemori H, Bae MA, Yazawa K, Sasaki T, Kobayashi J. (1992). Alteramide A, a new tetracyclic alkaloid from a bacterium Alteromonas sp. associated with the marine sponge Halichondria okadai. J Org Chem 57: 4317–4320.

[bib131] Shnit-Orland M, Kushmaro A. (2009). Coral mucus-associated bacteria: a possible first line of defense. FEMS Microbiol Ecol 67: 371–380. 19161430 10.1111/j.1574-6941.2008.00644.x

[bib132] Smith JE, Shaw M, Edwards RA, Obura D, Pantos O, Sala E et al. (2006). Indirect effects of algae on coral: algae‐mediated, microbe‐induced coral mortality. Ecol Lett 9: 835–845. 16796574 10.1111/j.1461-0248.2006.00937.x

[bib133] Stewart FJ, Ottesen EA, DeLong EF. (2010). Development and quantitative analyses of a universal rRNA-subtraction protocol for microbial metatranscriptomics. ISME J 4: 896–907. 20220791 10.1038/ismej.2010.18

[bib134] Struve C, Forestier C, Krogfelt KA. (2003). Application of a novel multi-screening signature-tagged mutagenesis assay for identification of Klebsiella pneumoniae genes essential in colonization and infection. Microbiology 149: 167–176. 12576590 10.1099/mic.0.25833-0

[bib135] Sunagawa S, DeSantis TZ, Piceno YM, Brodie EL, DeSalvo MK, Voolstra CR et al. (2009). Bacterial diversity and White Plague Disease-associated community changes in the Caribbean coral Montastraea faveolata. ISME J 3: 512–521. 19129866 10.1038/ismej.2008.131

[bib136] Sutherland KP, Shaban S, Joyner JL, Porter JW, Lipp EK. (2011). Human pathogen shown to cause disease in the threatened eklhorn coral Acropora palmata. PloS One 6: e23468. 21858132 10.1371/journal.pone.0023468 PMC3157384

[bib137] Torres AG, Redford P, Welch RA, Payne SM. (2001). TonB-dependent systems of uropathogenic Escherichia coli: aerobactin and heme transport and TonB are required for virulence in the mouse. Infect Immun 69: 6179–6185. 11553558 10.1128/IAI.69.10.6179-6185.2001 PMC98749

[bib138] Torto-Alalibo T, Collmer CW, Gwinn-Giglio M. (2009). The Plant-Associated Microbe Gene Ontology (PAMGO) Consortium: community development of new Gene Ontology terms describing biological processes involved in microbe-host interactions. BMC Microbiol 9: 1. 19278549 10.1186/1471-2180-9-S1-S1 PMC2654661

[bib139] Tout J, Jeffries TC, Petrou K, Tyson GW, Webster NS, Garren M et al. (2015). Chemotaxis by natural populations of coral reef bacteria. ISME J 9: 1764–1777. 25615440 10.1038/ismej.2014.261 PMC4511932

[bib140] Vandecandelaere I, Nercessian O, Segaert E, Achouak W, Mollica A, Faimali M et al. (2008). Alteromonas genovensis sp. nov., isolated from a marine electroactive biofilm and emended description of Alteromonas macleodii Baumann et al. 1972 (Approved Lists 1980). Int J Syst Evol Microbiol 58: 2589–2596. 18984698 10.1099/ijs.0.65691-0

[bib141] Vega Thurber R, Burkepile DE, Fuchs C, Shantz AA, McMinds R, Zaneveld JR. (2014). Chronic nutrient enrichment increases prevalence and severity of coral disease and bleaching. Glob Chang Biol 20: 544–554. 24277207 10.1111/gcb.12450

[bib142] Vega Thurber R, Payet JP, Thurber AR, Correa AM. (2017). Virus-host interactions and their roles in coral reef health and disease. Nature Rev Microbiol 15: 205–216. 28090075 10.1038/nrmicro.2016.176

[bib143] Vega Thurber R, Willner‐Hall D, Rodriguez‐Mueller B, Desnues C, Edwards RA, Angly F et al. (2009). Metagenomic analysis of stressed coral holobionts. Environ Microbiol 11: 2148–2163. 19397678 10.1111/j.1462-2920.2009.01935.x

[bib144] Voss JD, Richardson LL. (2006). Nutrient enrichment enhances black band disease progression in corals. Coral Reefs 25: 569–576.

[bib145] Vu-Khac H, Miller KW. (2009). Regulation of mannose phosphotransferase system permease and virulence gene expression in Listeria monocytogenes by the EIItMan transporter. Appl Environ Microbiol 75: 6671–6678. 19734332 10.1128/AEM.01104-09 PMC2772439

[bib146] Wear SL, Vega Thurber R. (2015). Sewage pollution: mitigation is key for coral reef stewardship. Ann N Y Acad Sci 1355: 15–30. 25959987 10.1111/nyas.12785 PMC4690507

[bib147] Weil E, Smith G, Gil-Agudelo DL. (2006). Status and progress in coral reef disease research. Dis Aquat Organ 69: 1–7. 16703761 10.3354/dao069001

[bib148] Weinbauer MG, Rassoulzadegan F. (2004). Are viruses driving microbial diversification and diversity? Environ Microbiol 6: 1–11. 14686936 10.1046/j.1462-2920.2003.00539.x

[bib149] Weynberg KD, Voolstra CR, Neave MJ, Buerger P, van Oppen MJ. (2015). From cholera to corals: viruses as drivers of virulence in a major coral bacterial pathogen. Sci Rep 5: 17889. 26644037 10.1038/srep17889 PMC4672265

[bib150] Whitworth T, Popov VL, Yu X-J, Walker DH, Bouyer DH. (2005). Expression of the Rickettsia prowazekii pld or tlyC gene in Salmonella enterica serovar Typhimurium mediates phagosomal escape. Infect Immun 73: 6668–6673. 16177343 10.1128/IAI.73.10.6668-6673.2005 PMC1230948

[bib151] Wiese J, Thiel V, Nagel K, Staufenberger T, Imhoff JF. (2009). Diversity of antibiotic-active bacteria associated with the brown alga Laminaria saccharina from the Baltic Sea. Mar Biotechnol 11: 287–300. 18855068 10.1007/s10126-008-9143-4

[bib152] Willis BL, Page CA, Dinsdale EA. (2004) Coral disease on the great barrier reef. Coral health and disease. Springer, New York, NY, USA, pp 69–104.

[bib153] Yoon J-H, Yeo S-H, Oh T-K, Park Y-H. (2004). Alteromonas litorea sp. nov., a slightly halophilic bacterium isolated from an intertidal sediment of the Yellow Sea in Korea. Int J Syst Evol Microbiol 54: 1197–1201. 15280291 10.1099/ijs.0.63079-0

[bib154] Yoshida K-i, Kobayashi K, Miwa Y, Kang C-M, Matsunaga M, Yamaguchi H et al. (2001). Combined transcriptome and proteome analysis as a powerful approach to study genes under glucose repression in Bacillus subtilis. Nucleic Acids Res 29: 683–692. 11160890 10.1093/nar/29.3.683 PMC30401

[bib155] Zhou C, Smith J, Lam M, Zemla A, Dyer MD, Slezak T. (2007). MvirDB—a microbial database of protein toxins, virulence factors and antibiotic resistance genes for bio-defence applications. Nucleic Acids Res 35: D391–D394. 17090593 10.1093/nar/gkl791 PMC1669772

