# Supplementary material for: Excess labile carbon promotes the expression of virulence factors in coral reef bacterioplankton
Source: ISME J. 2017 Sep 12;12(1):59–76. doi: 10.1038/ismej.2017.142 (PMC5739002; doi:10.1038/ismej.2017.142)
Supplement: Supplementray Information [file ismej2017142x1.docx]

Supplementary Discussion

*Energy-dependent regulation of virulence factor gene expression*

Based on the expression data from this study, we suggest two possible mechanisms of virulence factor regulation: The activation of global regulators linked to sugar catabolism and the activation of environmental sensing responses. Catabolism-dependent regulation relies on the PTS-dependent modulation of intracellular levels of the secondary messenger cyclic adenosine monophosphate (cAMP), via the activation of carbon catabolite repression (CCR) ([Notley-McRobb et al., 1997](#_ENREF_122)). As a consequence, high glucose levels are accompanied by low intracellular cAMP levels. The concentration of cAMP in the cell is a key determinant for the expression of a large number of genes, including catabolism of alternative sources of carbon and virulence. Virulence factors can be expressed under low ([Skorupski and Taylor, 1997](#_ENREF_149), [Kim et al., 2005](#_ENREF_84), [Wolfgang et al., 2003](#_ENREF_176)) and high ([Reverchon et al., 1997](#_ENREF_135)) cAMP intracellular levels. One example of such a sugar-dependent gene regulation in marine pathogens is the natural competence in *Vibrio cholera* induced by chitin and repressed by any preferred PTS-dependent sugars ([Blokesch, 2012](#_ENREF_14)). Levels of *cya* and *crp* genes encoding the adenylate cyclase and the cyclic AMP (cAMP) receptor protein (CRP), vary among the POPs with the different sugars (Table S5). Based on our results we speculate that GM_189 (*Alteromonas* sp.) and GM_554 (*Alcanivorax* sp.) possibly express virulence factors under low cAMP concentrations. We also found high induction of second messengers connected to carbon catabolism like bis-(3′-5′)-cyclic dimeric guanosinmonophosphate (c-di-GMP). Diguanylate cyclase activity (required for c-di-GMP synthesis) was induced under galactose in POPs GM_189 and MB_4 and under mannose in POPs GM_554 (*Alcanivorax* sp.), GM_66 and MB_1 (*Oceanicola* sp.). The switch between planktonic and attached lifestyles in several bacteria including *Roseobacter* clade members is balanced by this second messenger ([D'Alvise et al., 2014](#_ENREF_33), [Tischler and Camilli, 2004](#_ENREF_158), [Ueda and Wood, 2009](#_ENREF_162), [Hengge, 2009](#_ENREF_65)). This suggests sugar catabolism likely was an important regulator promoting settlement and virulence factor expression in POPs.

*Environmental sensing-dependent regulation of virulence factor gene expression*

The second mechanism suggested to be involved in virulence factor regulation is the activation of environmental sensing responses. An early environmental cue detected by bacteria after sugar enrichment may involve changes in the surrounding osmotic pressure. We found the prevalent induction of the two component systems for osmotic upshift sensing, EnvZ–OmpR, in the POPs. Under high solute concentrations, EnvZ–OmpR trigger water efflux and compatible solute accumulation ([Cai and Inouye, 2002](#_ENREF_21), [Sleator and Hill, 2002](#_ENREF_150)), but also act as a global regulator involved in the transcription of several proteins involved in pathogenesis, such as bacterial secretion system type III in *Salmonella typhimurium* ([Garmendia et al., 2003](#_ENREF_50)). Osmoregulatory mechanisms are also linked with the activation of bacterial motility and chemotaxis in a way that cells can be repelled under low osmolarity, attracted under optimal osmolarity and repelled under high osmolarity causing tumbling motility ([Li and Adler, 1993](#_ENREF_102), [Vaknin and Berg, 2006](#_ENREF_163)). In the same way, induction of chemotaxis was evidenced by the expression of several genes encoding for proteins involved in chemotaxis in all POPs (Table S5). It remains to be determined to what extent the pathways, identified here, are upregulated at sugar concentrations that are typically found in coral reefs exposed to wastewater outfalls ([Ziegler et al., 2016](#_ENREF_180)).

Another environmental factor commonly determining the regulation of virulence is low dissolved oxygen concentrations. Low dissolved oxygen concentrations can promote coral reef degradation by the increase of toxic substances, such as sulfide, a product of bacterial sulfate reduction and desulfuration in black band disease in corals ([Glas et al., 2012](#_ENREF_52)). We found an induction of genes involved in nitrate and sulfate reduction in the POPs (Table S5). These processes are prevalent in benthic and particle-attached bacteria, but not common in bacterioplankton communities. However, the increase in labile organic carbon results in increased bacterial respiration, creating microaerophilic environments suited for chemoheterothrophic activity, as previously reported on impacted reefs ([Costa Jr et al., 2000](#_ENREF_30), [Fabricius, 2005](#_ENREF_43)). Furthermore, we provided evidence for the induction of the sensor kinase MprB, a hypoxia-responsive regulator, linked to virulence expression in *Mycobacterium tuberculosis* ([He et al., 2006](#_ENREF_63)). MprAB directly participates in the expression of sigma factors including extracytoplasmic function (ECF) sigma factors SigE and SigB that in turn regulate the expression of numerous stress-responsive genes ([He et al., 2006](#_ENREF_63), [White et al., 2010](#_ENREF_172)).

We additionally found the induction of a few other regulators previously linked with virulence, such as PhoP-PhoQ, stimulated by Mg^2+^/Ca^2+^ ([Bijlsma and Groisman, 2005](#_ENREF_12)), RegX3-SenX3 with unknown environmental stimulus ([Parish et al., 2003](#_ENREF_125)), and elements of quorum sensing (Table S5) that might be involved in the expression of VF.


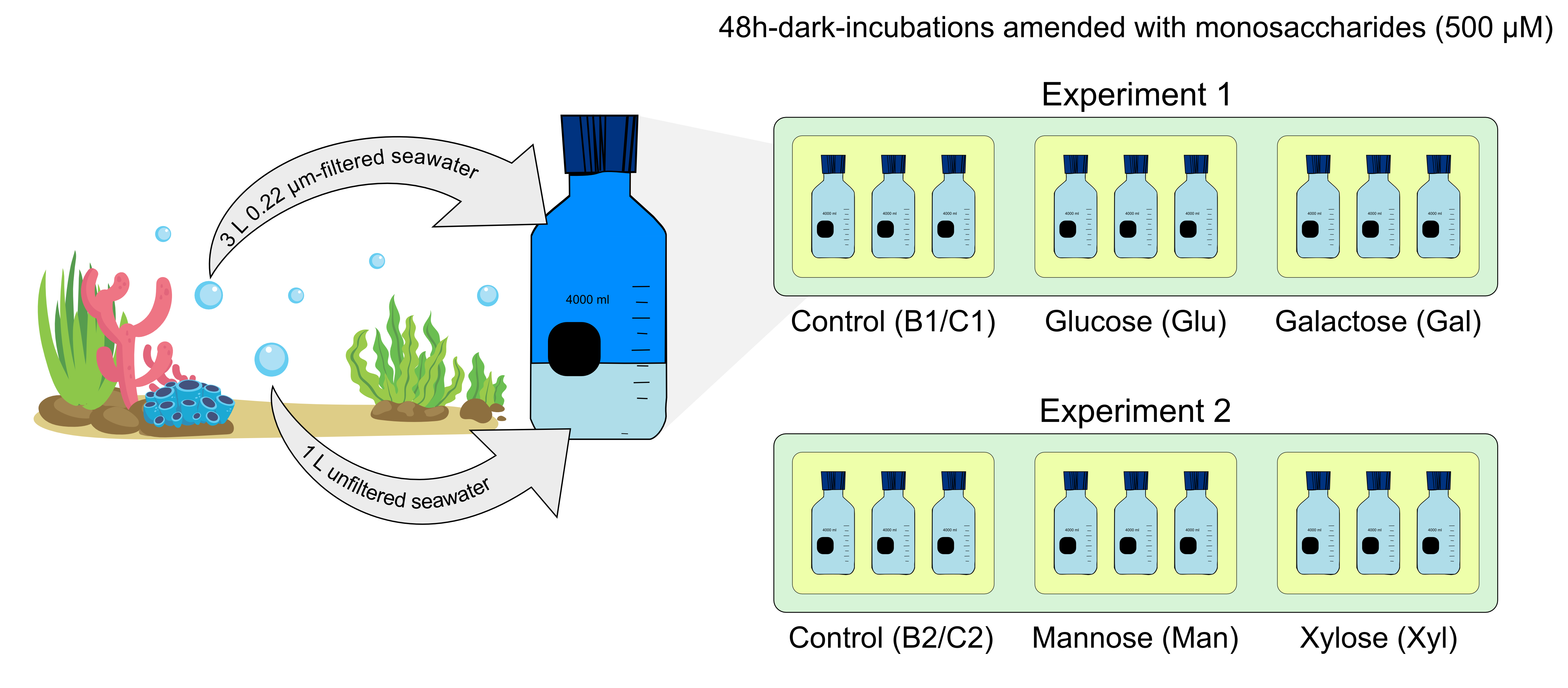


**Supplementary figure 1.** Schematic representation of the experimental setup: coral reef water amended with monosaccharides.


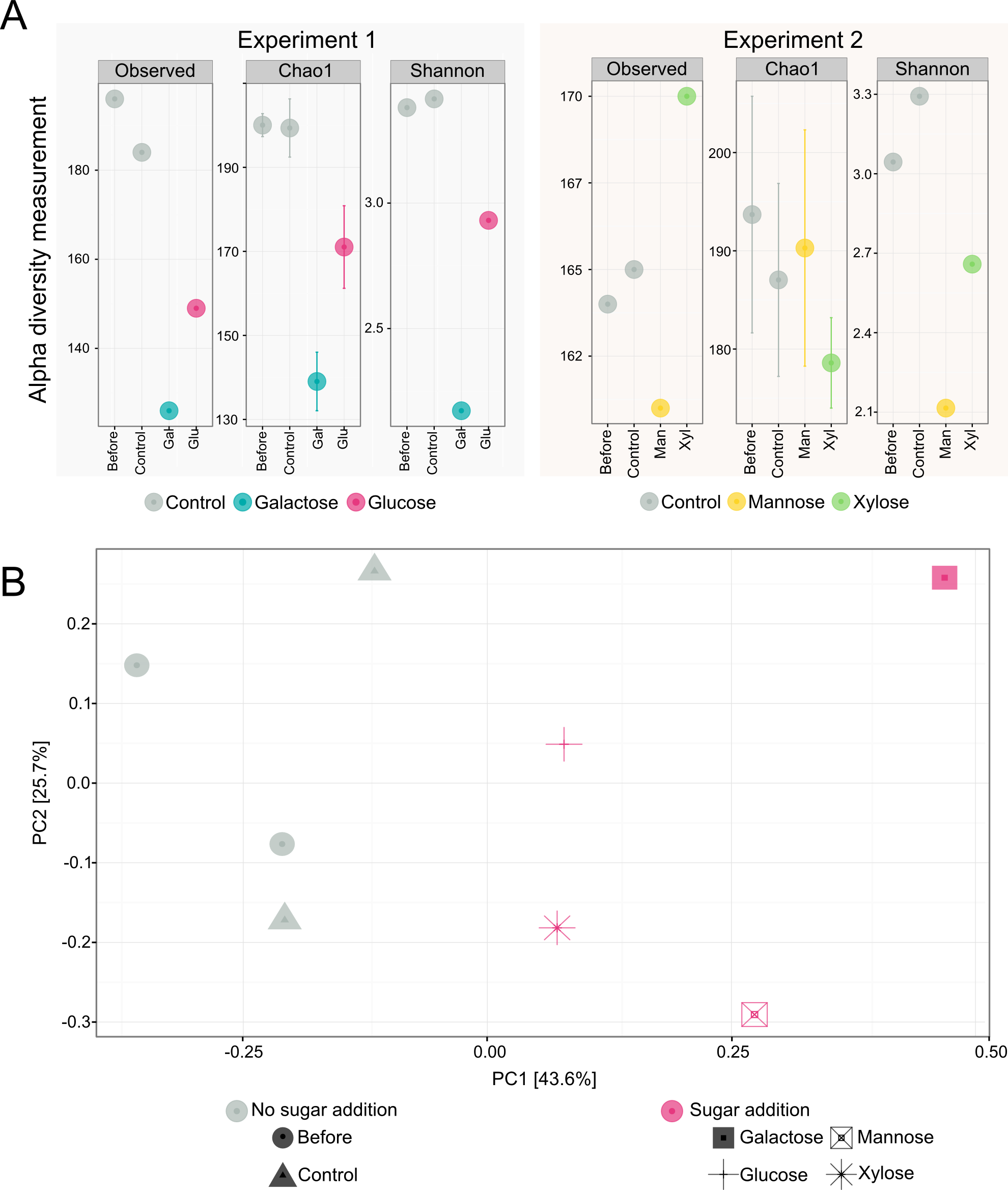


**Supplementary figure 2.** Alpha and beta diversity of reef microbial communities before and after sugar addition (A) Alpha diversity indices show a decrease in OTU richness and diversity after sugar additions. (B) Principal coordinates analysis plot based on Bray-Curtis distances between planktonic microbial reef communities. Sugar supplemented samples (magenta) have a different planktonic microbial community composition compared to untreated samples (grey), evidenced by a shift from left to right along PC1.


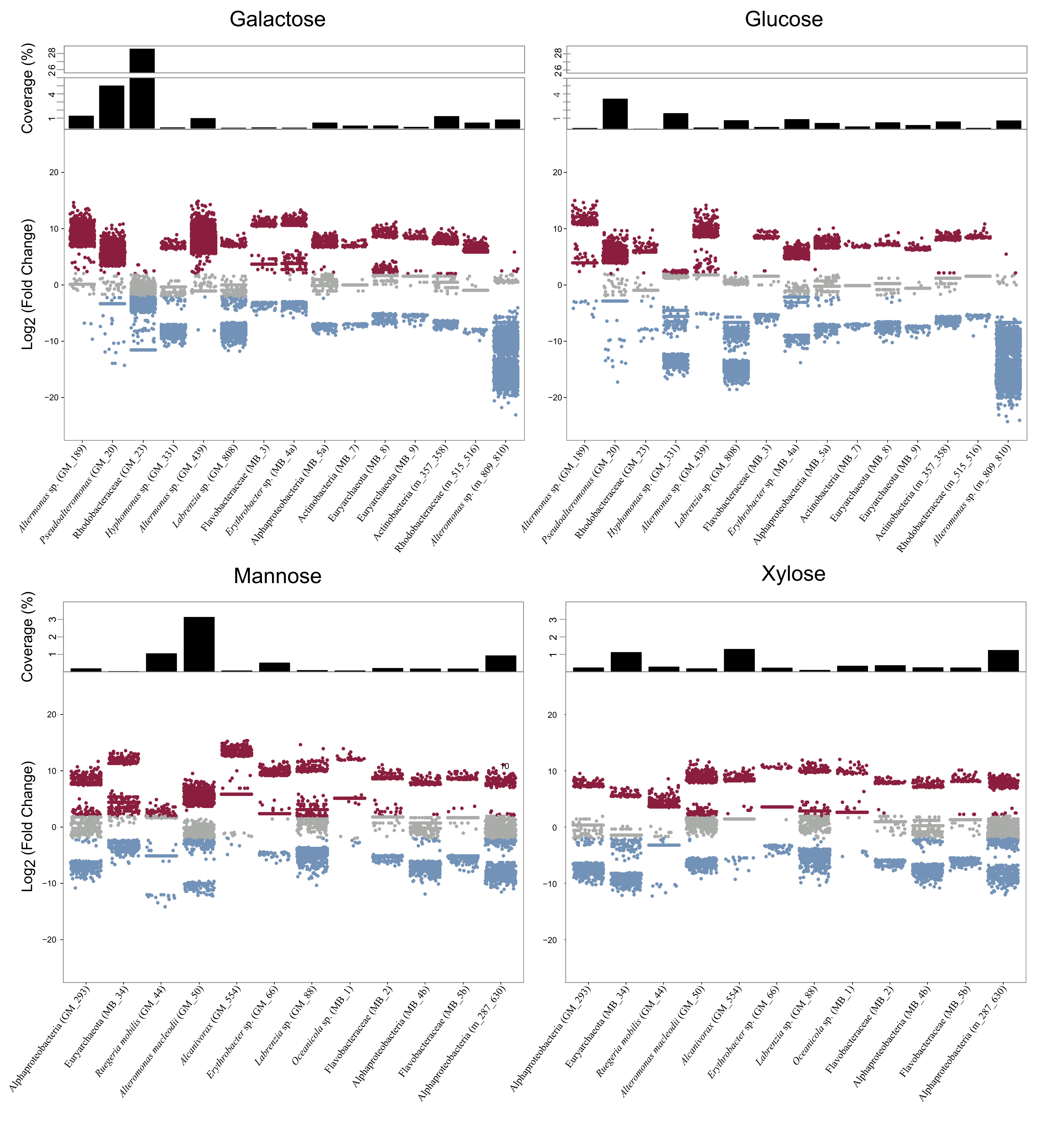


**Supplementary figure 3.** Gene expression patterns in microbial population genomes. Black bars denote bin abundance in the metagenome and dots represent log_2_ fold change comparing control and treatment of normalized mRNA counts. Log_2_ fold changes above 2 are colored in red and below -2 are colored in blue. Log_2_ fold change values between -2 and 2 are grey. Active populations are evidenced by a higher numbers of red dots.


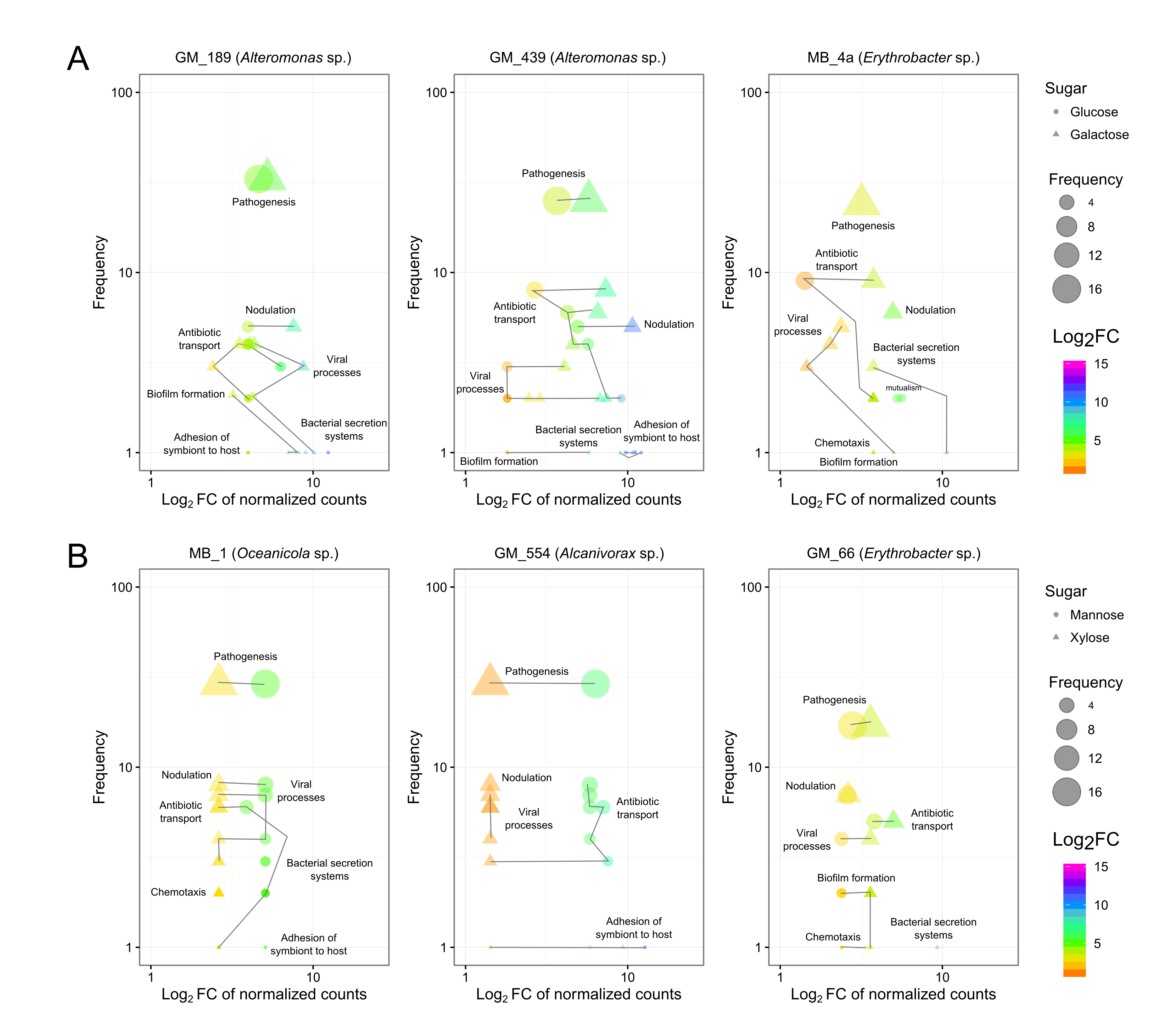


**Supplementary figure 4.** Expression of bacterial interaction genes in selected microbial population genomes. Interaction genes were clustered at the biological process (BP) level according to gene ontology (GO) annotation. Size denotes the number of genes in each group of interaction genes. Expression is represented in log_2_ fold change after comparing control and experimental treatments of normalized mRNA counts. Shapes correspond to different sugars in experiment 1 (A) and 2 (B).


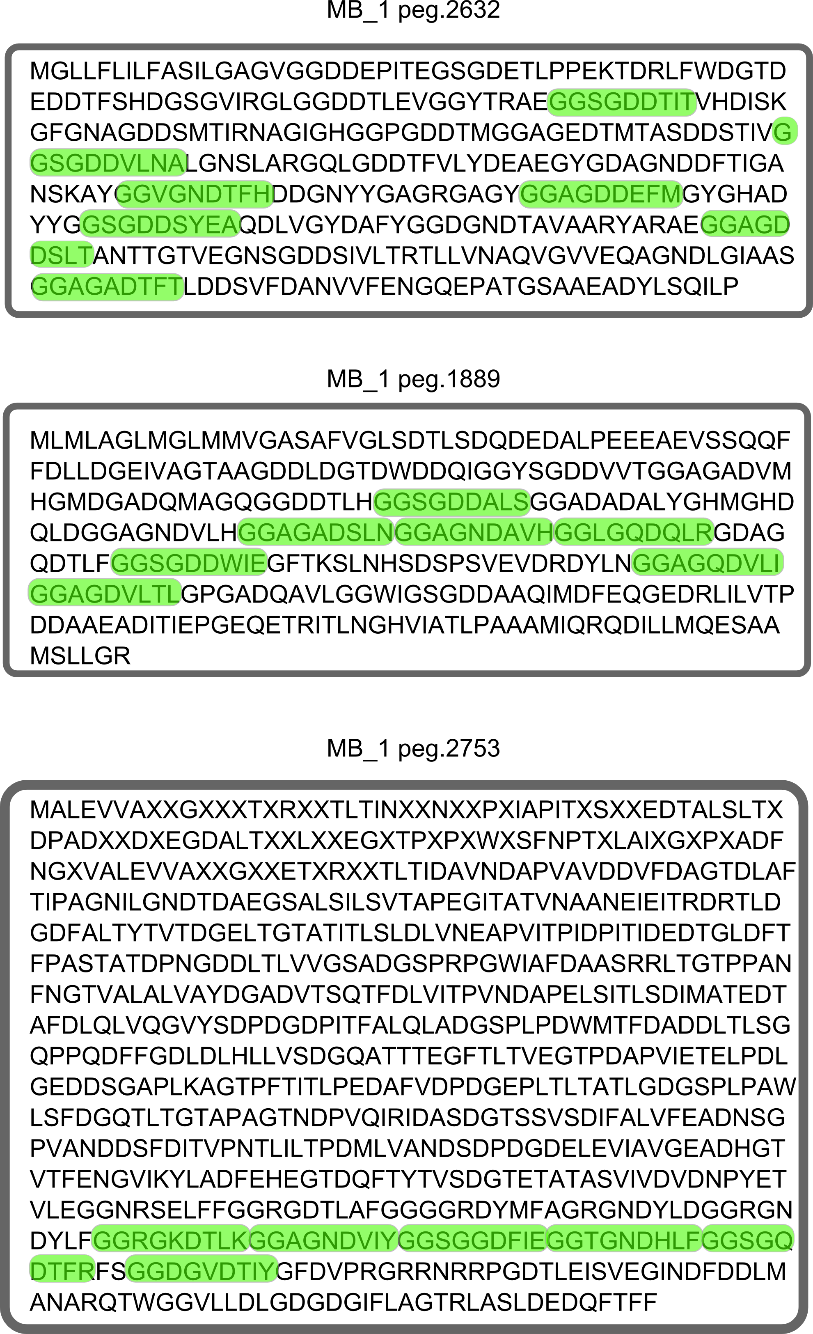


**Supplementary Figure 5.** Calcium-binding motifs of RTX homologs found in the population genome MB_1 (*Oceanicola* sp.).

**Supplementary table 1.** Relative abundance of coral reef bacterioplankton population genomes and number of reads mapped from metatranscriptomic libraries.

| **Population**  **genome** |  | **Relative abundance**  **(% metagenome)^*1^** | | |  | **Mapped normalized mRNA reads**  **(% metatranscriptome) ^*2^** | | |
| --- | --- | --- | --- | --- | --- | --- | --- | --- |
|  |  | **C1** | **Glu** | **Gal** |  | **C1** | **Glu** | **Gal** |
| GM_189 |  | 0,56 | 1,09 | 2,45 |  | 1298.98 (0.14) | 27837.94 (6.27) | 6893.58 (0.82) |
| GM_20 |  | 0,51 | 3,61 | 5,09 |  | 1267.52 (0.14) | 1150.62 (0.26) | 1973.36 (0.23) |
| GM_23 |  | <0,01 | 0,01 | 28,54 |  | 10890.96 (1.19) | 12128.17 (2.73) | 845.42 (0.1) |
| GM_331 |  | 0,03 | 1,87 | 0,04 |  | 70151.31 (7.63) | 613.43 (0.14) | 14296.27 (1.69) |
| GM_439 |  | 0,55 | 0,15 | 1,16 |  | 2020.78 (0.22) | 23936.25 (5.39) | 23557.46 (2.79) |
| GM_808 |  | <0,01 | 1,02 | <0,01 |  | 74299.01 (8.08) | 791.71 (0.18) | 86423.86 (10.24) |
| MB_3 |  | 0,63 | 0,21 | 0,04 |  | 349.14 (0.04) | 628.65 (0.14) | 6000.75 (0.71) |
| MB_4 |  | 0,26 | 1,15 | 0,01 |  | 2982.01 (0.32) | 1493.55 (0.34) | 44632.03 (5.29) |
| MB_5 |  | 0,58 | 0,69 | 0,62 |  | 1205.21 (0.13) | 1199.59 (0.27) | 1999.81 (0.24) |
| MB_7 |  | 0,25 | 0,27 | 0,26 |  | 228.97 (0.02) | 145.11 (0.03) | 314.62 (0.04) |
| MB_8 |  | 0,89 | 0,77 | 0,27 |  | 774.83 (0.08) | 211.59 (0.05) | 2934.35 (0.35) |
| MB_9 |  | 0,30 | 0,44 | 0,10 |  | 462.18 (0.05) | 255.87 (0.06) | 1352.63 (0.16) |
| merged_357_358 |  | 1,98 | 0,87 | 1,40 |  | 270.79 (0.03) | 403.15 (0.09) | 644.57 (0.08) |
| merged_515_516 |  | 0,31 | 0,10 | 0,62 |  | 221.19 (0.02) | 832.04 (0.19) | 1009.83 (0.12) |
| merged_809_810 |  | <0,01 | 0,99 | 0,99 |  | 1102.43 (0.12) | 8848.72 (1.99) | 8317.8 (0.99) |
|  |  |  |  |  |  |  |  |  |
|  |  | **C2** | **Man** | **Gal** |  | **C2** | **Man** | **Gal** |
| GM_293 |  | 0,29 | 0,17 | 0,22 |  | 7266.54 (0.48) | 11862.33 (2.61) | 2020.56 (0.32) |
| GM_34 |  | 0,41 | 0,01 | 1,10 |  | 4692.6 (0.31) | 40030.07 (8.82) | 370.86 (0.06) |
| GM_44 |  | 0,02 | 1,03 | 0,27 |  | 2375.17 (0.16) | 411.25 (0.09) | 3145.2 (0.51) |
| GM_50 |  | 0,40 | 3,11 | 0,17 |  | 27533.59 (1.83) | 7671.17 (1.69) | 57706.01 (9.28) |
| GM_554 |  | 1,44 | 1,05 | 3,28 |  | 163.29 (0.01) | 11907.7 (2.62) | 224.31 (0.04) |
| GM_66 |  | 0,66 | 1,50 | 1,21 |  | 369.77 (0.02) | 928.5 (0.2) | 160.45 (0.03) |
| GM_88 |  | 0,65 | 0,07 | 0,08 |  | 10029.28 (0.67) | 17616.44 (3.88) | 11040.33 (1.77) |
| MB_1 |  | 0,02 | 1,05 | 1,32 |  | 279.86 (0.02) | 1356.38 (0.3) | 659.4 (0.11) |
| MB_2 |  | 0,67 | 0,19 | 0,35 |  | 645.54 (0.04) | 1867.49 (0.41) | 637.39 (0.1) |
| MB_4 |  | 0,27 | 0,16 | 0,23 |  | 8496.67 (0.57) | 4361.19 (0.96) | 3020.51 (0.49) |
| MB_5 |  | 0,54 | 0,16 | 0,22 |  | 723.28 (0.05) | 1128.73 (0.25) | 708.95 (0.11) |
| merged_287_630 |  | 1,14 | 0,91 | 1,22 |  | 12254.48 (0.82) | 6252.62 (1.38) | 4738.6 (0.76) |

^*1^ Relative abundance of the population genomes was calculated as a proportion of metagenomic reads mapped to the genome from the total number of reads in the metagenomic library

^*2^ Number of mapped mRNA reads normalized by the size of the metatrancriptomic library and population genome relative abundance.

**Supplementary table 2.** Gene ontology (GO) terms used for functional annotation of microbial population genomes. Functional annotation included all child terms of the parental terms indicated in the table.

| GO term | Name | Used to infer |
| --- | --- | --- |
| GO:0004016 | *Adenylate cyclase activity* | cAMP synthesis |
| GO:0030552 | *cAMP binding* | cAMP receptor protein (CRP) |
| GO:0052621 | *Diguanylate cyclase activity* | Diguanylate cyclase synthesis |
| GO:0019419 | *Sulfate reduction* | Sulfate reduction |
| GO:0008940 | *Nitrate reductase activity* | Nitrate reduction |
| GO:0009372 | *Quorum sensing* | Quorum Sensing |
| GO:0044403 | *Symbiosis, encompassing mutualism through parasitism* | Bacterial interaction genes |
| GO:0006935 | *Chemotaxis* | Chemotaxis |
| GO:0009405 | *Pathogenesis* | Virulence factors |

**Supplementary table 3.** Taxonomic assignment of microbial population genomes using average nucleic acid identities (ANI), average amino acid identities (AAI), SpecI, PhyloPythiaS, and CheckM marker lineages.

| **Population genome** | **SpecI (marker gene identity)** | **CheckM marker lineage** | **PhyloPythiaS (assignments %)** | **Closest related species (ANI%\|AAI%)** |
| --- | --- | --- | --- | --- |
| GM_331 | *Hyphomonas neptunium* (79.73) | c__Alphaproteobacteria (UID3422) |  | *Hyphomonas neptunium* (71.58 \| 66.89) * |
| GM_189 | *Alteromonas* sp. SN2 (77.11) | c__Gammaproteobacteria (UID4761) |  | *Alteromonas macleodii* (68.37 \| 61.27) * |
| GM_23 | *Roseobacter* sp. SK209-2-6 (79.8) | f__Rhodobacteraceae (UID3360) |  | *Roseovarius nubinhibens* (69.36 \| 64.87) |
| MB_1 | *Oceanicola batsensis* (85) | f__Rhodobacteraceae (UID3361) |  | Oceanicola batsensis (77.80 \| 62.80) |
| MB_3 | Unassigned (n/a) | k__Bacteria (UID2569) | Actinobacteria (40.45) | *Robiginitalea biformata* (65.39 \| 45.85) |
| GM_439 | *Alteromonas* sp. SN2 (76.46) | c__Gammaproteobacteria (UID4761) |  | *Alteromonas macleodii* (69.03 \| 64.49) |
| MB_4a | *Erythrobacter* sp. SD-21 (85.09) | o__Sphingomonadales (UID3310) |  | *Erythrobacter citreus* (82.44 \| 70.44) |
| GM_293 | Unassigned (n/a) | c__Alphaproteobacteria (UID3305) |  | *Magnetospirillum magneticum* (63.76 \| 45.79) |
| GM_554 | *Alcanivorax* sp. DG881 (88.07) | c__Gammaproteobacteria (UID4443) |  | *Alcanivorax jadensis*(83.53 \| 80.34) * |
| merged_357_358 | Unassigned (n/a) | k__Bacteria (UID1453) | Actinobacteria (84.10) | *Gordonia paraffinivorans (65.58* \| 43.25) |
| GM_66 | *Erythrobacter* sp. SD-21 (86.56) | o__Sphingomonadales (UID3310) |  | *Erythrobacter citreus (83.32 \|*68.94) |
| GM_20 | *Pseudoalteromonas atlantica* (74.57) | c__Gammaproteobacteria (UID4761) |  | *Pseudoalteromonas spongiae* (65.58 \| 56.00) |
| GM_34 | Unassigned (n/a) | p__Euryarchaeota (UID3) |  | *Thermoplasmatales archaeon* (61.78 \| 35.27) |
| merged_809_810 | *Alteromonas macleodii* (82.1) | c__Gammaproteobacteria (UID4761) |  | *Alteromonas macleodii* (69.74 \| 64.46) |
| MB_2 | Unassigned (n/a) | k__Bacteria (UID2569) | Bacteroidetes (79.25) | *Zobellia_galactanivorans* (64.14 \| 45.51) |
| MB_5a | Unassigned (n/a) | c__Alphaproteobacteria (UID3305) |  | *Candidatus Puniceispirillum marinum* (62.24 \| 64.32) |
| GM_808 | *Labrenzia aggregata* (87.74) | o__Rhizobiales (UID3642) |  | *Labrenzia aggregata* (87.41 \| 91.22) |
| MB_4b | Unassigned (n/a) | c__Alphaproteobacteria (UID3305) |  | *Candidatus Puniceispirillum marinum* (68.46 \| 62.72) |
| GM_44 | *Silicibacter* sp. TrichCH4B (97.05) | f__Rhodobacteraceae (UID3375) |  | *Ruegeria mobilis* (97.27 \| 85.61) |
| MB_5b | Unassigned (n/a) | k__Bacteria (UID2569) | Actinobacteria (28.85) | *Robiginitalea biformata* (64.53 \| 35.91) |
| GM_50 | *Alteromonas macleodii* (93.69) | c__Gammaproteobacteria (UID4761) |  | *Alteromonas macleodii* (98.46 \| 98.00) |
| GM_88 | Unassigned (n/a) | c__Gammaproteobacteria (UID4444) |  | *Hahella ganghwensis* (66.08 \| 52.19) |
| MB_7 | Unassigned (n/a) | k__Bacteria (UID1453) | Actinobacteria (89.84) | *Acidimicrobium_ferrooxidans* (63.62 \| 35.96) |
| merged_515_516 | *Pelagibaca bermudensis* (78.79) | f__Rhodobacteraceae (UID3356) |  | *Dinoroseobacter shibae* (69.97 \| 62.89) |
| merged_287_630 | *Azospirillum* sp. B510 (70.61) | c__Alphaproteobacteria (UID3305) |  | *Candidatus Puniceispirillum marinum* (68.21 \| 63.64) |
| MB_8 | Unassigned (n/a) | p__Euryarchaeota (UID3) |  | *Archaeoglobus veneficus* (62.08 \| 35.78) |
| MB_9 | Unassigned (n/a) | p__Euryarchaeota (UID3) |  | *Archaeoglobus fulgidus* (61.40 \| 35.40) |

| **Virulence factor type** | **Gene name** | **Uniprot accession number** | **Contig ID** | **% of identity** | **length** | **e-value** | **bit score** | **Log2 fold change Glucose** | **Log2 fold change Galactose** | **Log2 fold change Mannose** | **Log2 fold change Xylose** |
| --- | --- | --- | --- | --- | --- | --- | --- | --- | --- | --- | --- |
| RTX toxin translocase | *hlyB* | P15492 | GM_189_peg.1875 | 41.11 | 343 | 8E-69 | 237 | 3.97 | 9.53 | n/a | n/a |
| RTX toxin translocase | *hlyB* | P15492 | GM_439_peg.3127 | 41.59 | 315 | 4E-57 | 206 | 1.81 | 8.93 | n/a | n/a |
| Cytotoxin | *tlyC* | O05961 | GM_66_peg.296 | 40 | 170 | 3E-32 | 123 | n/a | n/a | 2.39 | 3.60 |
| Cytotoxin | *tlyA* | P9WJ63 | GM_66_peg.498 | 44.12 | 102 | 3E-17 | 78.2 | n/a | n/a | 2.39 | 3.60 |
| Cytotoxin | *tlyC* | Q4UK99 | MB_1_peg.574 | 34.33 | 268 | 6E-42 | 152 | 3.97 | 0.10 | n/a | n/a |
| RTX toxin | *RtxA* | Q9KS12 | MB_1_peg.2632 | 31.27 | 291 | 2E-05 | 51.2 | n/a | n/a | 5.08 | 2.62 |
| RTX toxin | *LtxA* | P16462 | MB_1_peg.1889 | 36.5 | 200 | 8E-24 | 107 | n/a | n/a | 5.08 | 2.62 |
| RTX toxin | *frpC* | P55127 | MB_1_peg.2753 | 48.25 | 114 | 3E-19 | 97.4 | n/a | n/a | 5.08 | 2.62 |
| Zinc metalloprotease | *ZmpB* | Q9L7Q2 | MB_1_peg.1927 | 30.45 | 220 | 6E-09 | 61.6 | n/a | n/a | 5.08 | 2.62 |
| Zinc metalloprotease | *mcpb* | C5FYJ7 | GM_189_peg.2763 | 29.82 | 114 | 4E-06 | 52.4 | 3.97 | 7.92 | n/a | n/a |
| Protease | *cpc* | C5P1W9 | GM_189_peg.1834 | 35.51 | 107 | 1E-09 | 64.3 | 3.97 | 0.10 | n/a | n/a |
| Phosphatase | *ppx* | Q9ZN70 | GM_554_peg.2012 | 49.83 | 303 | 4E-97 | 302 | n/a | n/a | 5.85 | 1.42 |

**Supplementary table 4.** Extracellular proteases and toxins expressed in potential opportunistic pathogens (POPs)

**Supplementary table 5.** Log_2_ fold change of selected regulatory, metabolism, and chemotaxis genes.

|  | Adenilate cyclase activity | |  | cAMP-activated global transcriptional regulator CRP | |  | Diguanylate cyclase activity | |  | Chemotaxis | |
| --- | --- | --- | --- | --- | --- | --- | --- | --- | --- | --- | --- |
|  | Glucose | Galactose |  | Glucose | Galactose |  | Glucose | Galactose |  | Glucose | Galactose |
| GM_189 | 3.97 | 0.10 |  | 3.97 | 0.10 |  | 3.97 | 7.10 |  | 3.973 | 5.63 |
| GM_439 | 1.81 | 3.89 |  | 1.81 | -1.06 |  | 1.81 | -1.06 |  | 6.79 | 5.79 |
| MB_4 | 4.70 | 3.75 |  | n/a | n/a |  | -2.10 | 10.58 |  | 2.10 | 3.76 |
|  |  |  |  |  |  |  |  |  |  |  |  |
|  | Mannose | Xylose |  | Mannose | Xylose |  | Mannose | Xylose |  | Mannose | Xylose |
| MB_1 | 2.08 | 2.62 |  | n/a | n/a |  | 5.08 | 2.62 |  | 15.53 | 13.14 |
| GM_66 | 2.39 | 3.60 |  | n/a | n/a |  | 10.22 | 3.60 |  | 12.91 | 13.93 |
| GM_554 | 5.85 | 1.42 |  | 5.85 | 1.42 |  | 5.85 | 1.42 |  | 14.95 | n/a |

|  | Nitrate reduction | |  | Sufate reduction | |  | Acyl-homoserine lactone acylase/synthase | |  | Quinolone synthase | |
| --- | --- | --- | --- | --- | --- | --- | --- | --- | --- | --- | --- |
|  | Glucose | Galactose |  | Glucose | Galactose |  | Glucose | Galactose |  | Glucose | Galactose |
| GM_189 | 3.97 | 10.67 |  | n/a | n/a |  | 3.97 | 6.88 |  | 3.97 | 0.10 |
| GM_439 | 13.32 | 14.05 |  | 8.82 | 6.94 |  | 1.81 | 7.72 |  | 1.81 | 5.86 |
| MB_4 | n/a | n/a |  | 4.93 | 3.75 |  | -2.10 | 3.75 |  | n/a | n/a |
|  |  |  |  |  |  |  |  |  |  |  |  |
|  | Mannose | Xylose |  | Mannose | Xylose |  | Mannose | Xylose |  | Mannose | Xylose |
| MB_1 | 5.08 | 2.62 |  | n/a | n/a |  | 5.08 | 2.62 |  | n/a | n/a |
| GM_66 | n/a | n/a |  | 2.39 | 3.60 |  | n/a | n/a |  | n/a | n/a |
| GM_554 | 5.85 | 8.49 |  | n/a | n/a |  | 5.85 | 1.42 |  | n/a | n/a |
